# Fungi Contribute Critical but Spatially Varying Roles in Nitrogen and Carbon Cycling in Acid Mine Drainage

**DOI:** 10.3389/fmicb.2016.00238

**Published:** 2016-03-03

**Authors:** Annika C. Mosier, Christopher S. Miller, Kyle R. Frischkorn, Robin A. Ohm, Zhou Li, Kurt LaButti, Alla Lapidus, Anna Lipzen, Cindy Chen, Jenifer Johnson, Erika A. Lindquist, Chongle Pan, Robert L. Hettich, Igor V. Grigoriev, Steven W. Singer, Jillian F. Banfield

**Affiliations:** ^1^Department of Earth and Planetary Science, University of California, BerkeleyBerkeley, CA, USA; ^2^US Department of Energy Joint Genome InstituteWalnut Creek, CA, USA; ^3^Oak Ridge National LaboratoryOak Ridge, TN, USA; ^4^Graduate School of Genome Science and Technology, University of Tennessee-Oak Ridge National LaboratoryKnoxville, TN, USA; ^5^Earth Sciences Division, Lawrence Berkeley National LaboratoryBerkeley, CA, USA; ^6^Department of Environmental Science, Policy, and Management, University of California, BerkeleyBerkeley, CA, USA

**Keywords:** fungi, metagenomics, transcriptomics, proteomics, carbon, nitrogen, biofilm

## Abstract

The ecosystem roles of fungi have been extensively studied by targeting one organism and/or biological process at a time, but the full metabolic potential of fungi has rarely been captured in an environmental context. We hypothesized that fungal genome sequences could be assembled directly from the environment using metagenomics and that transcriptomics and proteomics could simultaneously reveal metabolic differentiation across habitats. We reconstructed the near-complete 27 Mbp genome of a filamentous fungus, *Acidomyces richmondensis*, and evaluated transcript and protein expression in floating and streamer biofilms from an acid mine drainage (AMD) system. *A. richmondensis* transcripts involved in denitrification and in the degradation of complex carbon sources (including cellulose) were up-regulated in floating biofilms, whereas central carbon metabolism and stress-related transcripts were significantly up-regulated in streamer biofilms. These findings suggest that the biofilm niches are distinguished by distinct carbon and nitrogen resource utilization, oxygen availability, and environmental challenges. An isolated *A. richmondensis* strain from this environment was used to validate the metagenomics-derived genome and confirm nitrous oxide production at pH 1. Overall, our analyses defined mechanisms of fungal adaptation and identified a functional shift related to different roles in carbon and nitrogen turnover for the same species of fungi growing in closely located but distinct biofilm niches.

## Introduction

Fungi are extremely diverse and abundant. They perform many critical ecosystem functions including the decomposition of organic matter, carbon storage, nutrient transfer, metal transformation and accumulation, and soil formation. The role of fungi in these ecosystem processes has been extensively studied using techniques such as cultivation, gene surveys, and enzyme or rate measurements. These approaches commonly target individual biological processes (e.g., carbon degradation). However, the full metabolic potential of fungi—across all of their genes, pathways, and processes—is rarely captured in an environmental context.

The full metabolic potential of environmentally-relevant organisms can be accessed by reconstructing genomes directly from environmental DNA sequences. The genome-centric metagenomics approach (involving sequence assembly) simultaneously recovers intact genomic sequences from many community members, thereby enabling analysis of organismal interactions. Specific functions can be connected to individual community members from reconstructed genomes. In fact, it is possible to recover complete and essentially complete bacterial and archaeal genomes from metagenomics datasets (e.g., Iverson et al., 2012; Wrighton et al., 2012; Albertsen et al., 2013; Castelle et al., 2013; Di Rienzi et al., 2013; Kantor et al., 2013; Sharon and Banfield, 2013). However, genome-centric metagenomics has rarely been applied to obtain intact eukaryotic genomes from environmental samples (Cantu et al., 2011; Cissé et al., 2012; Quandt et al., 2015), in part due to their larger, more complex, and often repeat-rich genomes.

Other genomic approaches have been successfully applied to microbial eukaryotes, but each of these approaches has limitations. Approximately 600 fungal genomes have been sequenced, but most of these are cultured representatives (which are not always environmentally relevant) and they only represent about half of known fungal diversity. Targeted metagenomics approaches (which use cell sorting coupled with multiple displacement amplification, MDA), have successfully sequenced partial eukaryotic genomes (Cuvelier et al., 2010; Yoon et al., 2011; Monier et al., 2012; Vaulot et al., 2012); however, MDA reactions lead to inherent biases in genome coverage (Woyke et al., 2010). Read-based genomic sequencing (which maps reads to known genomes using similarity searches such as BLAST) has provided some functional information from root-associated fungi (Liao et al., 2014), but this approach is limited by genome availability and incomplete genome annotations. It remains to be seen if recent advances in sequencing technologies and assembly approaches make metagenomics more feasible for recovery of fungal genomes from mixed communities.

Expression of predicted genes can be measured at the level of mRNAs and proteins as a proxy for cellular activity in the environment. Metatranscriptomic and metaproteomic techniques allow for the quantification of thousands of transcripts and proteins from individual taxonomic groups within mixed communities. Combining transcriptomic and proteomic measurements allows for the integration of diverse molecular-level processes to predict system-level function.

Here, we applied a combination of genome-centric metagenomics, transcriptomics and proteomics methodologies to test for differentiation of fungal metabolism as a function of microbial community context in a well-characterized microbial ecosystem, acid mine drainage (AMD) biofilms from the Richmond Mine (Iron Mountain near Redding, California). Fungi are found in two different types of biofilms within the mine: (1) the widespread floating biofilms (characterized extensively with metagenomics and proteomics in prior studies), and (2) the less common streamer biofilms. Floating biofilms are found at the air-AMD solution interface and are dominated by *Leptospirillum* Group II bacteria, which fix carbon into the system and support the growth of other heterotrophic microbes (e.g., (Denef et al., 2010) and references therein). Many complex sugars have been previously shown to be constituents of the extracellular matrix in the floating biofilms (Jiao et al., 2010). Streamer biofilms, dominated by fungi, grow as long filaments submerged within the flowing AMD solution and are anchored to pyrite sediments within the stream (Baker and Banfield, 2003). Very little is known about streamer biofilm communities, or about the physiology and ecology of the prolific fungal members.

Floating and streamer biofilm are found in close proximity to each other and experience essentially the same bulk AMD solution (extremely acidic, pH <1; metal-rich, ~200 mM Fe; and thermophilic, 40–50°C) and underlying pyrite sediment. Nonetheless, fungal abundance is very different in the two biofilm types: fungi dominate streamer biofilm communities, but are found in low abundance in floating biofilms (Baker and Banfield, 2003; Baker et al., 2004, 2009). We hypothesize that variations in fungal metabolism between the biofilm types influence the observed distribution patterns. For instance, fungi likely play a role in degrading organic carbon within both biofilms, but the carbon sources may vary between the floating biofilms and the submerged, streamer biofilms.

## Materials and methods

### Biofilm sample collection

AMD biofilms were collected from the Richmond Mine (Iron Mountain near Redding, California) into sterile tubes that were flash-frozen in an ethanol/dry ice bath on-site before transferring to a −80°C freezer upon return to the laboratory. The streamer biofilms used for community genome sequencing were sampled at the 5-way site (28Feb08). Streamer biofilms used for initial mRNA sequencing for gene model prediction and annotation were sampled from the 4-Way site (09Jun09). For expression studies, two different types of AMD biofilms were collected: (1) floating biofilms, which are found at the air-AMD solution interface, and (2) streamer biofilms, which are submerged within the AMD solution and are anchored to pyrite sediments within the stream. Floating biofilms (characterized as mature, growth stage two biofilms based on biofilm thickness) were sampled from the AB-Muck site on three different dates: 17Sept10 (F1); 21Oct11 (F2); and 28Dec11 (F3). Streamer biofilms were sampled on three different dates at two sites: 4-Way site, 17Sept10 (S1); 4-Way site, 21Oct11 (S3); AB+10m site, 02Nov12 (S4). All of the sampling locations are within ~100 meters of each other, with essentially the same underlying pyrite sediment and AMD solution with low pH (typically 0.5–1.2), elevated temperature (30–56°C), and millimolar concentrations of sulfate, iron, zinc, copper, and arsenic (Druschel et al., 2004).

### Microscopy and fluorescence *in situ* hybridization

Differential interference contrast (DIC) and epifluorescent microscopy was carried out at 630X magnification on a standard epifluorescence microscope. Fluorescence *in situ* hybridization (FISH) was carried out on fixed (4% paraformaldehyde) AMD biofilm samples as described previously (Amann et al., 1995; Bond and Banfield, 2001; Baker et al., 2009). Oligonucleotide probes used in this study for identification of individual species and groups were as follows: EUBMIX (all Bacteria); ARC915 (all Archaea); EUKMIX (all Eukaryotes); DOH299 (*Dothideomycetes* Class); LF655 (all *Leptospirillum* bacteria); LF1252 (*Leptospirillum* group III bacteria); L2UBA353 (*Leptospirillum* group II UBA genotype); L2CG353 (*Leptospirillum* group II 5-way genotype); and SUL230 (*Sulfobacillus* spp.). A total of 1045–1506 cells were counted for each biofilm sample, from 3 to 6 fields of view per probe (with an average of 313 cells counted per probe per sample). Counts were converted to a percentage of the total cell count found using the general nucleic acid stain 4′,6-diamidino-2-phenylindole (DAPI). Images for figures were adjusted for contrast and combined with GIMP (www.gimp.org).

### *A. richmondensis* culture growth conditions

*Acidomyces richmondensis* strain BFW was isolated from streamer biofilms collected at the 5-way location of the Richmond Mine on 28Feb08 that were frozen on dry ice at the site and stored at –80°C. The biofilms were thawed and homogenized (in a glass tube by using several vigorous strokes of a tight-fitting, round, glass pestle), and then inoculated into minimal media (pH 1) containing glucose and ampicillin, as previously described (Baker et al., 2004). After growth was observed, the mycelia were spread on potato dextrose agar plates (Becton Dickinson, Sparks, MD). Individual fungal colonies were re-streaked after 1 week of growth. One of these colonies was then regrown in the liquid medium. Culture purity was verified by microscopy and PCR amplification and sequencing of 16S and 18S ribosomal RNA genes. This *A. richmondensis* isolate has been maintained at 4°C for ~4 years.

To test for the production of nitrous oxide (N_2_O) gas, *A. richmondensis* was grown in sealed serum vials with a modified M9 minimal medium at pH ~1 (6.78 g/L Na_2_HPO_4_; 3 g/L KH_2_PO_4_; 0.5 g/L NaCl; 1 g/L NH_4_Cl; 2 mM MgSO_4_; 0.1 mM CaCl_2;_0.4% glucose; 2 g/L NaNO_3_; 0.034 mg/ml chloramphenicol). Triplicate cultures were grown under high and low oxygen conditions. For high oxygen conditions, the media and headspace were aerobic but the supply of air was discontinued after the serum vials were sealed. For low oxygen conditions, the aerobic culture was inoculated into anaerobic vials (flushed with ultrapure nitrogen gas) at a 0.01 volume/volume ratio. After 6 days of growth, gas samples from the headspace were analyzed for N_2_O on a Shimadzu GC-14A gas chromatograph fitted with a ^63^Ni electron capture detector.

### DNA extraction

Samples for genomic DNA sequencing (both from biofilms and cultures) were thawed in cold 0.9% NaCl, pH 1, by manual homogenization with a sterile, cut-off 1000 μL pipette tip. Homogenized samples were centrifuged at 7000 × g for 5 min at 4°C, decanted, and resuspended in 1 mL 0.9% NaCl, pH 1. A 16-gauge needle was used to further homogenize the biofilm. The homogenate was centrifuged at 7000 × g for 5 min at 4°C, decanted, and resuspended in 1 mL 1X PBS, pH 7. A 16-gauge needle was used to further homogenize the biofilm, which was then centrifuged at 7000 × g for 5 min at 4°C. The supernatant was decanted, and the remaining pellet was flash frozen in liquid nitrogen, and transferred to a pre-chilled, sterile mortar and pestle. The cell pellet was ground to a fine powder, adding liquid N_2_ as necessary to keep the powder frozen. Powder was optionally stored for up to a month in liquid nitrogen before proceeding. Frozen powder was thawed in 1 mL pre-warmed CTAB buffer, pH 8, with 1% β-mercaptoethanol and 1% 0.5M EDTA. Samples were incubated at 70°C for 1 h, with occasional mixing by hand and three freeze/thaw cycles in liquid N_2_ and a 70°C heat block after 30 min. An equal volume (1 mL) of phenol:chloroform:isoamyl alcohol (25:24:1) was added to a pre-spun phase lock gel heavy 15 mL tube (5 PRIME, Gaithersburg, MD), and samples were centrifuged for 5 min at 1500 × g at room temperature after hand mixing. A second 1 mL of phenol:chloroform:isoamyl alcohol was added to the tube, mixed, and centrifuged as before. A final equal volume of chloroform:isoamyl alcohol (24:1) was added and centrifuged as before. The aqueous supernatant was removed, and DNA was precipitated with 1X volume of isopropanol and 0.1X volume 3M NaOAc (pH 5.8) at −20°C for at least 1 h, pelleted and washed with 70% EtOH, and resuspended in 50 μL TE pH 8.

### *A. richmondensis* culture genomic sequencing and assembly

The *A. richmondensis* BFW isolate genome was sequenced using 454/Roche pyrosequencing (454-Rapid, 1 full run, 590.7 Mbp) and Solexa/Illumina sequencing (GAIIx, 3 lanes, “2 × 76, 300 bp” insert, 6.4 Gbp total sequencing). DNA (500 ng) was fragmented by nebulization to an average size of 500–800 bp and size selected using SPRI beads (Beckman Coulter, Indianapolis, IN). The fragments were treated with end repair and ligated with adapters using the 454™ Rapid Library preparation kit (Roche, Basel, Switzerland) and then sequenced. DNA (1 μg) was sheared using the Covaris E210 (Covaris, Woburn, MA) and gel-size selected for 300 bp. The fragments were treated with end-repair, A-tailing, and ligation of Illumina compatible adapters (IDT, Coralville, IA) followed by 10 cycles of PCR (NEB, Ipswich, MA). qPCR was used to determine the concentration of the libraries, according to JGI standard operating procedures using the KAPA Complete Library Quantification Kit (Kapa Biosystems, Wilmington, MA). Libraries were sequenced on the Illumina Hiseq. Data were quality-control filtered for artifacts and process contamination. Illumina reads were first assembled with velvet (Zerbino and Birney, 2008) version 0.7.55 (*k* = 61, -min_contig_lgth 100 -exp_cov 59 -cov_cutoff 15). The resulting contigs were shredded to 1000 bp fragments with 800 bp overlaps. Additionally, 400 bp reverse complement fragments were created from each contig terminus. These shredded contigs and the 454 reads were subsequently assembled with Newbler (www.454.com) version 2.5 (-info -consed -nrm -finish -rip -sio -a 50 -l 350 -g -ml 20 -mi 97 -e 17). Mitochondrial sequences were screened out and assembled separately.

### Metagenomic-based sequencing and assembly of *A. richmondensis*

The *A. richmondensis* genome was also assembled using metagenomic sequencing reads (Aciri1_meta assembly). One library (mean insert size 193 bp) was prepared and sequenced on three lanes (2 × 76 bp) of an Illumina GAIIx flowcell and one lane (2 × 150 bp) of an Illumina HiSeq2000 flowcell using standard protocols at the US Department of Energy Joint Genome Institute. Raw reads are deposited at the National Center for Biotechnology Short Read Archive under study ID SRP006615. For the GAIIx runs, reads were trimmed with an in-house script that removed bases from the 3′ end of reads until a base with a PHRED quality score of ≥3 was found. Only read pairs with both reads ≥43 bp after trimming were kept for further processing. Overlapping HiSeq reads were merged and adapter sequences removed using SeqPrep (https://github.com/jstjohn/SeqPrep) with the following parameters: -6 -m 0.3 -n 0.7 -o 12 -Z 100000 -N 1.

Assembly was performed in stages designed to gradually select and assemble reads likely to be from *A. richmondensis*. Initially, the merged reads from the HiSeq library above were assembled using velvet (Zerbino and Birney, 2008) version 1.1 with the columbus module (*k* = 99, -cov_cutoff 5.0 -exp_cov 19.7 -ins_length 193 -ins_length_sd 27.4) using a bowtie v.1 (Langmead et al., 2009) mapping of reads to the isolate genome as a reference. Contigs greater than three kbp from this assembly were binned based on tetranucleotide content using emergent self-organizing maps (ESOM) (Dick et al., 2009). Contigs 2–3 kbp in length were then added to the ESOM by calculating their tetranucleotide frequencies and projecting these 2–3 kb contigs onto best match positions on the fixed ESOM learned from the set of contigs greater than three kbp in length, using the Project command in Databionics ESOM Tools (http://databionic-esom.sourceforge.net). Contigs belonging to a large, obvious fungal bin in the ESOM were manually selected and exported. Putative fungal contigs were initially identified based on membership in one or more of the following contig sets. Set (1) contained contigs in the manually identified fungal ESOM bin. Set (2) contained contigs that were nearby to contigs in set (1) in the genome assembly graph (≤two edges away in velvet's final de Bruijn graph, defined using velvet's LastGraph file). That is, these contigs showed some read-based evidence of contiguity with contigs in set (1) or evidence of contiguity with contigs that were first neighbors to contigs in set (1). Set (3) contained contigs with a best blast hit of any putative protein on the contig to a fungal protein with *e* ≤ 1e-10. All reads from all sequencing runs were mapped against this set of putative fungal contigs with bowtie as unpaired reads, and all mapping reads (and paired reads, if available) were collected. Velvet was again used to assemble this subset of reads using the isolate genome as a mapped reference (*k* = 75, -cov_cutoff 5.0 -exp_cov 20.5 -ins_length 193 -ins_length_sd 27.4). Contigs were split at any run of ambiguous N bases (there are no scaffolds in this assembly), and used as input to a new ESOM-based binning. Contigs were selected from the resulting large fungal bin, and searched against an in-house database of known AMD bacteria and archaea from the study site, as well as draft *Sulfobacillus* genomes assembled from this data. Based on this, 12 contigs were removed from the assembly as potential non-fungal contaminants. After data analysis, one additional contig (01615) was identified as having similarity to a new *Sulfobacillus* genome. Finally, contigs were broken at potential mis-assemblies using an in-house script that looks for regions with zero paired-read support, as previously described (Sharon et al., 2013).

Assemblies were annotated using a standard pipeline implemented in the MycoCosm fungal web portal at the JGI (Grigoriev et al., 2012, 2014). Prediction of filtered gene models was aided by 24.4 Gbp of initial cDNA sequencing (Illumina HiSeq) derived from poly-A selected streamer community RNA.

For comparison, we also assembled the metagenomic reads without using information from the isolate assembly, following an analogous iterative protocol to that above. Velvet was used to assemble the merged HiSeq library reads as above, without the aid of the reference, and contigs ≥3 kb from the resulting assembly was binned via ESOM-based binning of tetranucleotide frequencies. Contigs between 1000 and 2999 bp were added to the ESOM map by projection. The ESOM structure, BLAST hits to an in-house database of known AMD bacteria and archaea from the study site, and BLAST hits to *Dothideomycete* genomes were used to identify the large putative fungal bin in the assembly. All reads from all sequencing runs were mapped to contigs from this selected bin and contigs in the DeBruijn assembly graph within two edges of these contigs. Mapped reads and their read pairs were used as input to a new velvet assembly (*k* = 75, -cov_cutoff 5.0 -exp_cov 24.5 -ins_length 193 -ins_length_sd 27.4). Contigs were split at any run of N bases, and a new ESOM was prepared with contigs ≥3 kb, with contigs 1000–2999 bp then projected on the map. The fungal bin was manually selected based on ESOM topography and BLAST hits as above. Selected contigs were manually screened via BLAST against the in-house database of AMD organisms and against the NCBI nr database for non-fungal contigs, and 38 contigs were removed. Lastly, contigs were quality-screened as above for misassemblies.

### Genome completeness

Genome completeness was estimated based on the presence of conserved, low-copy-number genes in both the isolate and metagenome assemblies of *A. richmondensis*. CEGMA (Parra et al., 2009) version 2.5 was run with default parameters, and completeness was estimated based on the presence of partial or complete hits to 248 “core eukaryotic genes.” We also considered hits only to “group 4” genes, which represent the most conserved KOGs. In addition, the “clusters” tool in MycoCosm (Grigoriev et al., 2012, 2014) was used to identify all gene family clusters (based on Markov clustering of predicted proteins; Enright et al., 2002) that exist in single copy in the 50 other available *Dothideomycete* genome assemblies available (May 2014). We then counted the number of these clusters that contained one or more representative proteins from the isolate or metagenome assemblies of *A. richmondensis*.

### *A. richmondensis* functional analyses

MycoCosm (Grigoriev et al., 2012, 2014) was used to evaluate gene annotations for the *A. richmondensis* genome, including InterPro domain (Hunter et al., 2009), KOG assignment (Tatusov et al., 1997, 2003), GO terms (Ashburner et al., 2000), PFAM domains (Punta et al., 2011), KEGG categories (Kanehisa et al., 2008), and BLAST hits (Altschul et al., 1990).

Carbohydrate Active Enzymes (CAZymes) were predicted using sequence libraries that were built with full length and constitutive modules (glycoside hydrolase, GH; polysaccharide lyase, PL; carbohydrate esterase, CE; glycosyltransferase, GT; and carbohydrate-binding module, CBM) isolated from the collection of carbohydrate-active enzymes of the CAZy database (www.cazy.org) (Cantarel et al., 2009). A series of profile hidden Markov models (HMMs) were built from each of the module families (and subfamilies in a number of cases) described by the CAZy database. Assignment of the protein models to a CAZy family (or to several families in the case of multimodular proteins) was performed by a two-step procedure that involved first a BLAST (Altschul et al., 1997) search against the full length CAZy proteins, keeping for further analysis all proteins that gave a *e*-value smaller than 0.1. The selected proteins were then subjected to a combination of BLAST and HMMer3 (Eddy, 2009) searches against the libraries of sequence made with the CAZy modules and the collection of HMM profiles, respectively. All positive results giving significant scores with both BLAST and HMMs were manually inspected, checked for the presence of catalytic residues or sequence motifs characteristic of the family, annotated for possible problems (for instance, if the model was a fragment), and assigned to one or several CAZy families.

### Molecular phylogeny

The intergenic transcribed spacer region (ITS) of the *A. richmondensis* genome was identified via BLAST (Altschul et al., 1997) against a set of previously identified *Dothideomycetes* fungi (Yamazaki et al., 2010), followed by manual adjustment. Fungal strains with identical ITS sequences were removed from the set, and sequences were aligned with mafft using default nucleotide parameters (mafft-linsi) (Katoh and Standley, 2013). Gblocks (Talavera and Castresana, 2007) was used to trim the alignment to conserved positions in minimum block-lengths of five nucleotides that were deemed informative for phylogenetic analyses. This resulted in an alignment of 424 positions in 19 conserved blocks. FastTree (Price et al., 2010) was used for approximate maximum likelihood tree inference with support values under the discrete gamma model with 20 rate categories (–gamma). All *Bispora* sp. MEY-1 coding sequences (HM003045.1, HM003044.1, HM003043.1, GU351880.1, GU074519.1, FJ695140.1, FJ492963.1, FJ472925.1, FJ212324.1, EU919724.1) were downloaded from the NCBI nucleotide database, and searched against both assemblies with default blastn parameters.

### RNA extractions

Biofilm samples were prepared for RNA extractions by resuspending 1 mL of frozen biofilm (from a 2 mL microcentrifuge tube) in 1 mL 1X PBS (pH 7), centrifuging at 15,000 rpm for 2 min at 4°C, resuspending in 1 mL 1X PBS (pH 7), and then splitting the resulting suspension into separate aliquots of ~100 μL. Aliquots were pelleted by centrifugation, cleaned of supernatant and immediately flash frozen on liquid nitrogen and stored at −80°C until processing, within 1 month of preparation. Prior to RNA extraction, 1 mL of RLC Buffer (from the RNeasy Plant Mini Kit, QIAGEN, Valencia, CA) with 10% (v/v) 2-mercaptoethanol (2-ME) was added to each pellet. The resulting mixture was transferred to Lysing Matrix E bead beating tubes (MP Biomedicals, Santa Ana, CA) and immediately flash frozen in liquid nitrogen. Tubes were then thawed and bead beaten for 30 s using a Fast Prep-24 Automated Homogenizer (MP Biomedicals, Santa Ana, CA) at 6.0 m/s. Following bead beating, tubes were flash frozen again. Tubes were then heated for 1 min at 65°C, vortexed for 30 s, heated at 65°C for 2 min, vortexed for 30 s and briefly centrifuged to settle cell debris. Five hundred microliters of the resulting supernatant was processed using the QIAGEN RNeasy Plant Mini Kit (QIAGEN, Valencia, CA) following the manufacturer's protocol.

RNA extraction replicates were pooled (10–40 replicates per biofilm sample) and then treated with DNase using the QIAGEN RNase-free DNase Set, following the manufacturer's protocol (QIAGEN, Valencia, CA). Samples were further cleaned and concentrated with the RNeasy MinElute Cleanup Kit (QIAGEN, Valencia, CA). Pooled, cleaned and concentrated samples were assessed for purity, quality, and concentration using a NanoDrop Spectrophotometer (NanoDrop Products, Wilmington, DE), Qubit Broad Range RNA Assay (Life Technologies, Grand Island, NY), and an Agilent Bioanalyzer with the RNA 6000 Nano Kit (Agilent Technologies, Santa Clara, CA), yielding 23–187 μg RNA per biofilm sample.

### RNA sequencing

Total RNA was processed and sequenced by the Joint Genome Institute. Two separate approaches were used to enrich mRNA in the biofilm samples: (1) poly-A selection to target eukaryotic transcripts, and (2) ribosomal RNA (rRNA) depletion using the RiboZero human and bacteria kits (Epicentre) for targeting transcripts from the entire community. Transcripts were converted to cDNA and sequenced with an Illumina HiSeq 2000 instrument using 150 base pair (bp), paired-end reads.

Reads were trimmed to 36 bp and then aligned with BWA [seed length = 25, maximum hits = 1; (Li and Durbin, 2009)] to an in-house reference database containing nearly 80,000 predicted transcripts derived from previous genomic characterizations of cultures and biofilms from the Richmond Mine AMD system (Denef et al., 2010 and references within). Reads from the poly-A selected biofilm samples were aligned to the *A. richmondensis* (Aciri1_meta) transcriptome available in MycoCosm (Grigoriev et al., 2012, 2014). The number of reads that aligned to each gene were counted for each sample, only considering uniquely mapping reads and those mapping to one strand.

### Protein extraction

AMD biofilm samples were prepared for protein extractions by removing 100–200 mg of frozen biomass and centrifuging at 15,000 rpm at 4°C for 2 min to pellet biofilm and remove any residual fluid. Proteins were extracted using a protocol modified from previously reported methodology (Chourey et al., 2010). Briefly, cells were lysed using three approaches. First, cell pellets were flash frozen with liquid nitrogen and ground with a micropestle. Ground pellets were then resuspended in 1 mL SDS cell lysis buffer (5% SDS; 50 mM tris-HCl, pH 8; 150 mM NaCl; 0.1 mM EDTA; 1 mM MgCl_2_) with 12 μL 4 M dithiothrietol. This solution was transferred to a Lysing Matrix E bead beating tube (MP Biomedicals, Santa Ana, CA) and processed for 30 s using a Fast Prep-24 Automated Homogenizer (MP Biomedicals, Santa Ana, CA) at 6.0 m/s. Tubes were centrifuged briefly and the supernatant was transferred to 15 mL tubes. This solution was then sonicated using an Ultrasonic Processor (QSonica, Newtown, CT) for three intervals of 1 min at 100 amps followed by 1 min of rest. Next, the lysed cell solution was incubated for 15 min at 99°C, cooled, vortexed for 3 min and centrifuged at 10,000 rpm for 10 min at 4°C. Pelleted cell debris was discarded and 300 μL 100% trichloroacetic acid (TCA) was added to the supernatant cell lysates which were then incubated overnight at 4°C to precipitate proteins. Following overnight incubation, precipitated proteins were pelleted by centrifuging at 14,000 rpm for 20 min at 4°C. Pellets were washed three times with 1 mL acetone, centrifuging at 14,000 rpm for 10 min at 4°C between each wash. Once washed, the pellet was allowed to air dry and then resuspended in guanidine HCl buffer (6 M guanidine HCl; 50 mM Tris, pH = 7.6; 10 mM CaCl_2_). Total protein concentrations were estimated with the bicinchoninic acid (BCA) assay (Pierce BCA Protein Assay Kit; Thermo Scientific, Waltham, MA).

### Proteomics methods

Each protein sample was reduced with DTT (10 mM, final concentration). Fifty micrograms of proteins from each AMD biofilm sample was further processed with the Filter-Aided Sample Preparation (FASP) method (Wiśniewski et al., 2009) following the manufacturer's protocol (Expedeon, San Diego, CA). Proteins were digested overnight with sequencing grade trypsin (Promega, Madison, WI) in an enzyme:substrate ratio of 1:100 (wt:wt) at room temperature with gentle shaking, followed by a second digestion for 4 h with the same amount of trypsin (i.e., 0.5 μg). The peptide samples were eluted from the filter by centrifugation and then stored at −80°C.

### 2D-LC-MS/MS proteomic measurements

The multi-dimensional protein identification technology (MudPIT) (Washburn et al., 2001) was used in our analytical workflow. In each MudPIT run, 50 μg of peptides were loaded offline into a 150-μm-I.D. 2D back column (Polymicro Technologies, Phoenix, AZ) packed with 3 cm of C18 reverse phase (RP) resin (Luna, Phenomenex, Torrance, CA) and 3 cm of strong cation exchange (SCX) resin (Luna, Phenomenex, Torrance, CA). The back column was connected to a 100-μm-I.D. front column (New Objective, Woburn, MA) packed in-house with 15 cm of C18 RP resin. The back column and front column were placed in-line with a U3000 quaternary HPLC pump (Dionex, Sunnyvale, CA). Prior to the measurement, the back column loaded with peptides was de-salted offline with 100% Solvent A (95% H_2_O, 5% CH3CN, and 0.1% formic acid), and washed with a 1 h gradient from 100% Solvent A to 100% Solvent B (30% H_2_O, 70% CH3CN, and 0.1% formic acid) to move peptides from RP resin to SCX resin. Each MudPIT run was configured with the 11 SCX-RP separations in 22 h. 5, 7, 10, 12, 15, 17, 20, 25, 35, 50, and 100% of Solvent D (500 mM ammonium acetate dissolved in Solvent A) were used in the 11 SCX fractionations. Each SCX fraction was separated by a 110 min RP gradient from 100% Solvent A to 60% Solvent B.

The AMD biofilm samples were measured on an LTQ Orbitrap Velos Pro mass spectrometer (Thermo Scientific, Waltham, MA). MS scans were acquired in Orbitrap with the resolution of 30,000. After each MS scan, the top 20 most abundant precursor ions were selected for fragmentation by collisional-induced dissociation with 35% normalized collision energy (NCE) and 10 ms activation time. Fragment ions were measured in ion trap. Precursor ions with unassigned charge states and +1 were rejected for MS/MS analysis. Dynamic exclusion was enabled with ±10 parts-per-million (ppm) exclusion window width and 60 s of exclusion duration.

### Protein identification and quantification

All MS/MS spectra were searched with Sipros (Pan et al., 2011; Hyatt and Pan, 2012; Wang et al., 2013) against a database containing ~80,000 proteins derived from ~80 Gb of genomic information obtained from previous genomic characterizations of biofilms sampled from the Richmond Mine AMD system. The database was concatenated with reverse sequences for estimation of false discovery rate (FDR), using the following search parameters: parent mass offsets of −1, 0, +1, +2, +3 Da; 0.03 Da and 0.5 Da of mass tolerances for parent ions and fragment ions, respectively; up to three missed cleavages; full enzyme specificity required; dynamic modifications included oxidation of methionine and deamidation of asparagine and glutamine; static modification included carbamidomethylation of cysteine.

The raw search result of each MudPIT run was filtered individually to achieve 1% FDR at the peptide level estimated by concatenated reverse peptide sequence identifications. Proteins were inferred from the identified peptides using parsimony rules. Briefly, indistinguishable proteins were combined into protein groups and subset proteins and subsumable proteins were removed. A minimum of two peptides, at least one of which must be unique, was required for each inferred protein or protein group, which resulted in <1% FDR at the protein level, estimated by concatenated reverse protein sequence identifications.

Protein quantification was carried out with ProRata (Pan et al., 2006; Wang et al., 2013). For protein quantification via spectrum counting, spectrum counts of proteins were balanced by taking the sum of the spectrum counts uniquely mapping to a gene plus a fraction of the non-unique spectra split between matching proteins.

### Proteome- and transcriptome-based community structure analyses

For the community-level analysis of the biofilms, transcript read counts, and protein spectral counts were normalized by making the total “library-size” of each sample identical. Transcript read counts from the ribosomal RNA-depleted libraries were normalized by the sum of all mapped paired-end reads in the sample and multiplied by 1,000,000. Balanced protein spectrum counts were normalized by the sum of all balanced spectrum counts in the sample and multiplied by 1,000,000. After normalization, only uniquely mapping proteins (to one protein from one organism) were considered for further analyses.

For organism-level analysis of the biofilms, protein spectral counts were further normalized by making the total sum of an individual organism (e.g., *A. richmondensis*) identical across all samples. Poly-A selected RNA was used for *A. richmondensis* organism-level transcriptome analyses, normalized in the same manner.

Hierarchical clustering was performed on transcript and protein abundance values normalized at the community-level and organism-level (for *A. richmondensis*) with absolute intensities converted to percentages for each transcript/protein (the sum of the percentages for each transcript/protein is equal to 100%). The clustering method used an uncentered Pearson correlation distance matrix and average linkage clustering (using Multi-experiment Viewer; MeV_4_8; www.tm4.org/mev/) (Saeed et al., 2003). For clustering analyses only, 0.000001 was added to each number to avoid software adjustments of zero values.

Community structure was evaluated by summing the transcript read counts and protein spectral counts for each organismal group (e.g., *Leptospirillum* group III, archaea, etc.) and then dividing by the total sum of all transcripts/proteins in the sample (using community-level normalized values).

### Differential expression analyses

For differential expression analyses, transcripts and proteins were only considered if they were quantified in all five biofilm replicates. Differential expression of individual transcripts and proteins were identified as those with abundance ratios (e.g., floating biofilm:streamer biofilm) >2 or <0.5 combined with a Rank Product *p* = 0.01, similar to methods used in other studies (Williamson et al., 2008; Dobbin et al., 2010; Soares et al., 2010; Zhao et al., 2010; Han et al., 2011; Jain et al., 2011; Muthukrishnan et al., 2011). Rank Product, commonly used in microarray experiments, is a non-parametric statistical method based on ranks of fold changes (Breitling et al., 2004). In the significance analyses, transcripts and proteins that are considered as up-regulated in one condition are concomitantly considered as down-regulated under the other condition. Counts were not normalized by gene/transcript/protein length in order to minimize data transformations, thus, only transcripts/proteins with the largest fold change or greatest significance between treatments were considered, rather than the largest overall abundance in a sample.

Gene Set Enrichment Analysis (GSEA) (Subramanian et al., 2005) was used to determine whether expression of defined set of genes (e.g., genes involved in amino acid biosynthesis) showed statistically significant differences between conditions (floating vs. streamer biofilms). Gene set analyses can illuminate important effects on overall pathways that might be missed by single-gene analyses. GSEA settings were: gene set permutation; classic enrichment analysis; and log_2_ ratio of classes metric for ranking genes.

### Accession numbers

The *A. richmondensis* genomic sequence assemblies have been deposited at DDBJ/EMBL/GenBank under the accession numbers JOOL00000000 (metagenome assembly) and JPDO00000000 (isolate assembly). Genomic and transcriptomic data is available in the MycoCosm web-portal (http://jgi.doe.gov/fungi; Project ID: 402991). The mass spectrometry proteomics data have been deposited to the ProteomeXchange Consortium via the PRIDE (Vizcaíno et al., 2016) partner repository with the dataset identifier PXD003541.

## Results

### Morphology of *A. richmondensis* in biofilms and in culture

**Figure 1:**
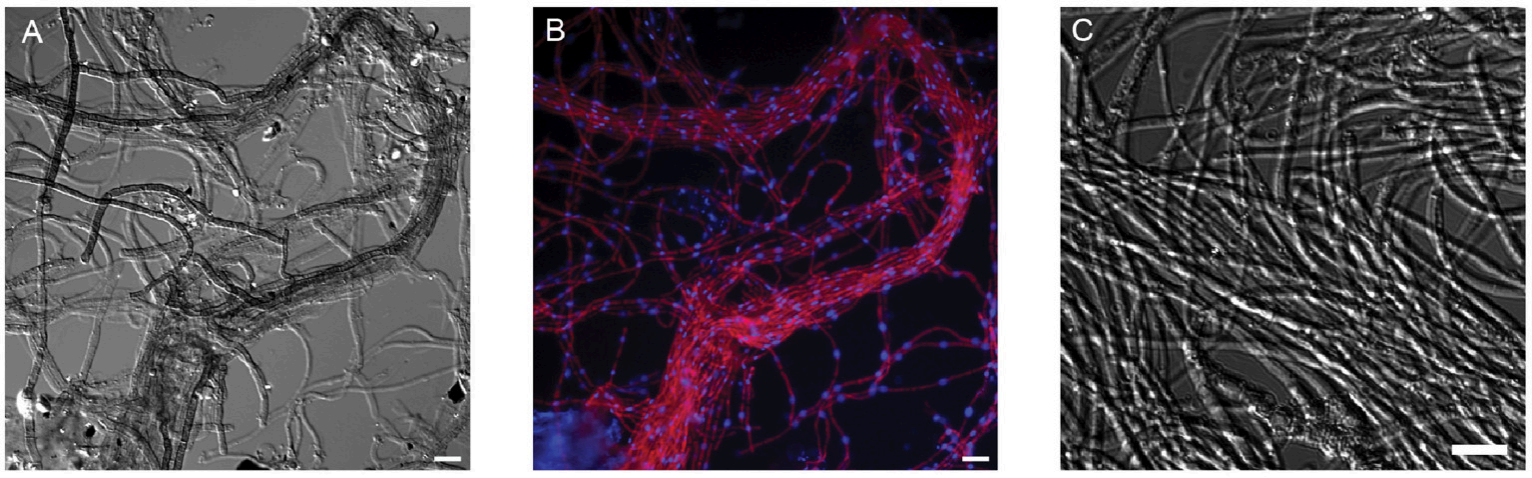
**Morphology of ***A. richmondensis*** from natural acid mine drainage streamer biofilm samples (A,B) and in pure culture (C). (A)** DIC microscopy of melanized hyphae in characteristic streamer bundles. **(B)** FISH microscopy of the same field of view as **(A)**; red is the DOH299-Cy3 probe for *Dothideomycetes*, blue is the general nucleic acid DAPI stain. **(C)** DIC microscopy of hyphae in pure culture. Scale bar in all panels is 10 μm.

Floating and streamer biofilms were collected from the Richmond Mine (Iron Mountain near Redding, California). In the context of prior molecular surveys (Baker et al., 2009), microscopy indicated that *A. richmondensis* was likely the only eukaryote in the biofilms sampled, based on fluorescence *in situ* hybridization with probes specific to *Eukaryotes* and to *Dothideomycetes* (Figure 1). *A. richmondensis* strains were previously observed in other biofilms from the mine, based on microscopy and phylogeny (Baker et al., 2004, 2009), but no prior studies have evaluated its genomic potential, physiology, or ecology.

In the streamer biofilms, *A. richmondensis* grew as dark hyphae in microscopic bundles, with very few branchings (Figure 1). Hyphae were ~2.7 μm wide, with septa spaced approximately every 16.5 μm. In floating biofilms, *A. richmondensis* grew as filaments interspersed within the biofilm matrix and did not form thick bundles as seen in the streamer biofilms.

### Assembly of *A. richmondensis* genome from metagenomic and genomic sequence datasets

Genome assembly was performed from Illumina and Roche/454 sequencing reads derived from an isolate of *A. richmondensis*. The *A. richmondensis* genome was also assembled using Illumina sequencing reads derived from the metagenome of a natural streamer biofilm community containing fungi, bacteria, archaea, and viruses. The metagenome reads were assembled in two ways: (1) using the assembled genome from the culture isolate as a reference, and (2) as a completely separate dataset independent of the culture isolate reads. All three approaches produced assemblies of comparable length and number of contigs (Table 1). The reference-guided metagenome assembly had the best overall contiguity so it was used for all downstream analyses. The assembly was 26.8 Mbp in length with 10,352 predicted genes, compared to the average *Dothideomycetes* genome of 38.9 Mbp with 11,955 genes (Supplemental Dataset) (Ohm et al., 2012).

**Table 1:** **Summary statistics for ***A. richmondensis*** genome assemblies for the isolate, the guided metagenome assembly (using the culture reference), and the independent metagenome assembly**.

**Sample**	**Isolate**	**Metagenome (guided)**	**Metagenome (independent)**
Sequencing technology	454, Illumina GAIIx	Illumina GAIIx, HiSeq	Illumina GAIIx, HiSeq
Total sequencing	2.4 Gbp	18.8 Gbp	18.8 Gbp
Assembled length^a^	29.3 Mbp	26.8 Mbp	26.1 Mbp
Contigs^a^	2254	1683	2122
N50 (bp)^a^	42,539	45,775	25,824
NG50 (bp)^a^^,^ ^b^	41,600	38,627	21,432
Largest contig (bp)	215,673	252,107	229,804
Predicted genes	11,202	10,352	N/A
Protein length (median)	333	353	N/A
Genes with intron	7300 (65.2%)	6746 (65.2%)	N/A
Intron length (median)	63	63	N/A
Exons per gene (mean)	2.28	2.3	N/A
BLAST hit to NR (NCBI) (% of total)	9071 (80.98%)	8767 (84.69%)	N/A
BLAST hit to Swissprot (% of total)	7186 (64.15%)	7068 (68.28%)	N/A
%GC	49.3%	49.7%	49.7%
Estimated completeness (Eukaryotic core genes)	96.8%	96.8%	97.2%
Estimated completeness (Dothideomycete core genes)	99.5%	99.4%	N/A

a *For contigs ≥ 1000 bp*.

b *N50 for assumed genome length of 30 Mbp*.

Two different approaches estimate high genome and predicted proteome completeness based on the presence of conserved genes (Table 1). First, we used the CEGMA pipeline (Parra et al., 2009) to estimate completeness based on the presence of 248 eukaryotic orthologous groups (KOGs) (Koonin et al., 2004) identified as present in single or low copy number across six diverse eukaryotic genomes. This CEGMA approach yielded estimates of 96.8% completeness, and 100% completeness when only a subset of the most conserved KOGs is considered. Second, genome completeness was estimated considering 1007 gene families (based on protein clustering) present in single copy within all of the other *Dothideomycete* genomes available in the MycoCosm fungal genome portal (Grigoriev et al., 2012, 2014). In the *A. richmondensis* metagenome assembly, 99.4% of these families were represented (97.7% of these were present as a single protein).

### Phylogenetic context for *A. richmondensis*

As closely related *Dothideomycetes* have not yet been sampled genomically, the assembled *A. richmondensis* internal transcribed spacer (ITS) region (Schoch et al., 2012) was used to place the fungus within a broader phylogenetic context (Supplemental Figure S1). *A. richmondensis* belonged to the *Dothideomycetes* class of the *Ascomycota* phylum. The assembled *A. richmondensis* ITS was 95% identical to *Acidomyces acidophilum*, an organism previously suggested to be identical to *A. richmondensis* that was isolated from acidic soils and mine drainage in Canada (as *Scytalidium acidophilum*) (Sigler and Carmichael, 1974), Northern Europe (Selbmann et al., 2008), and the Czech Republic (Hujslová et al., 2009). The assembled ITS region was identical to the recently described *Teratosphaeria acidotherma*, isolated from acidic Japanese hot springs (Yamazaki et al., 2010), and to *Bispora* sp. MEY-1, isolated from acidic uranium AMD in China (Luo et al., 2008). *T. acidotherma* (renamed as *Acidomyces acidothermus*) was also isolated from acidic soils in the Czech Republic and Iceland (Hujslová et al., 2013). Ten *Bispora* sp. MEY-1 carbohydrate-active enzyme genes each aligned to *A. richmondensis* contigs with 99–100% identity. Thus, *A. richmondensis* and closely related fungi appeared to be globally distributed in diverse acidic environments, and future genome sequencing will help resolve the molecular phylogeny of these organisms.

### Biofilm community transcriptome and proteome

Quantitative transcriptomics and shotgun proteomics were used to determine transcript and protein abundance and to infer function in three replicates each of floating and streamer biofilms. The floating biofilm sample F3 was removed from subsequent analyses because a low number of transcripts and proteins were recovered (45–46%) compared to the other five biofilm samples. Additionally, the community transcriptome was uncorrelated to any other sample (based on hierarchical clustering and Pearson correlation values ≤ 0.33). Taken together, this suggests that there may have been a technical error in the RNA and proteins extractions from the F3 biofilm, or that the biofilm matrix differed in some way, possibly resulting in extraction biases.

Across the five remaining biofilms, 55,829 different transcribed genes were quantified from 80,044,780 total paired-end reads (Supplemental Dataset). Poly-A selected transcriptomics libraries generated another 49,570,388 paired-end reads (Supplemental Dataset). A total of 7791 different proteins were quantified from the biofilm communities, 7004 of which could be uniquely assigned to a single protein in a single organism (Supplemental Dataset). Most of these proteins were quantified across multiple biofilm samples. From the 10,352 genes predicted in the *A. richmondensis* genome, a total of 10,260 transcripts and 2497 proteins were quantified in the biofilms.

**Figure 2:**
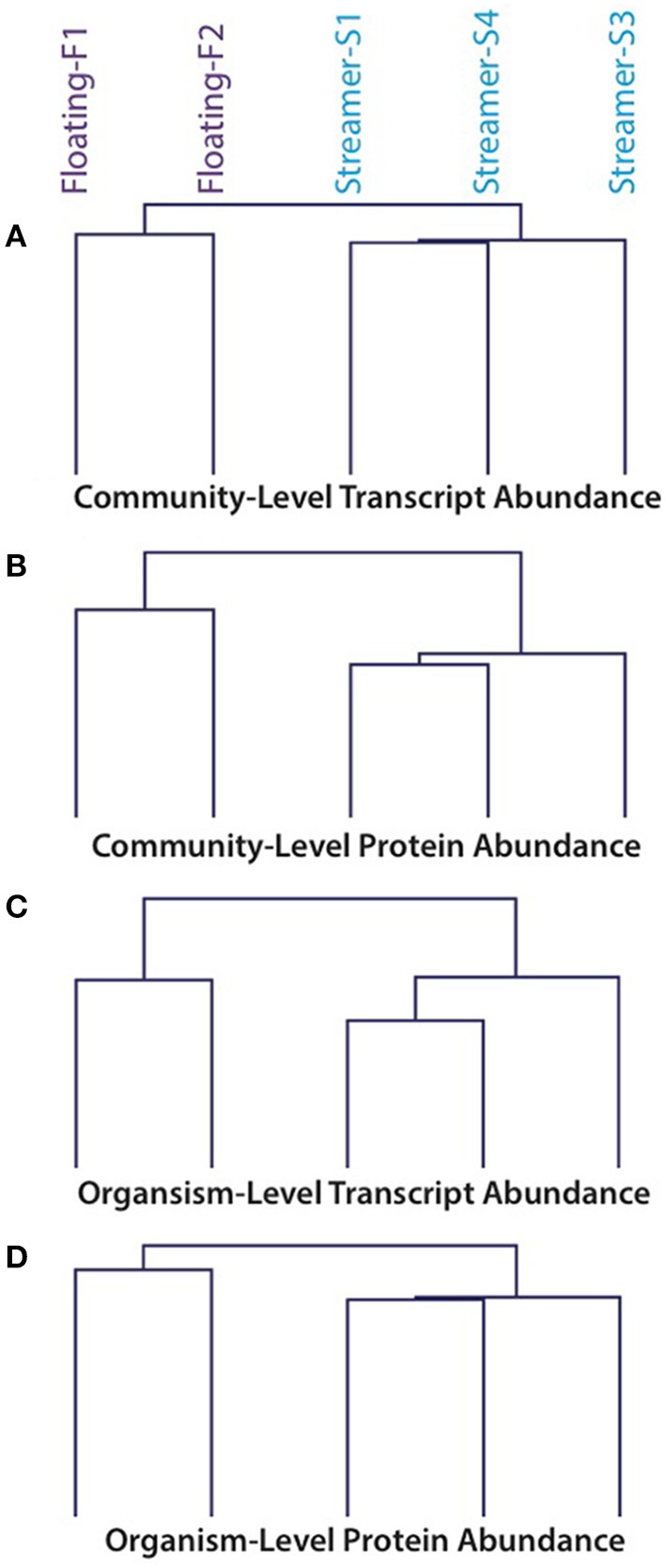
**Hierarchical clustering of transcript and protein abundance values from floating and streamer biofilms**. **(A)** Transcript abundance values normalized at the community-level. **(B)** Protein abundance values normalized at the community-level. **(C)** Transcript abundance values normalized at the organism-level for *A. richmondensis*. **(D)** Protein abundance values normalized at the organism-level for *A. richmondensis*.

Community transcriptomes and proteomes were strongly correlated within the same condition (floating or streamer biofilms) but not between different conditions (Pearson correlation coefficients: within floating 0.78–0.92; within streamer 0.85–0.93; between floating and streamer 0.14–0.35). Hierarchical clustering of transcript and protein abundance showed that floating and streamer biofilms clustered into separate groups (Figure 2).

### Community expression profile of floating and streamer biofilms

**Figure 3:**
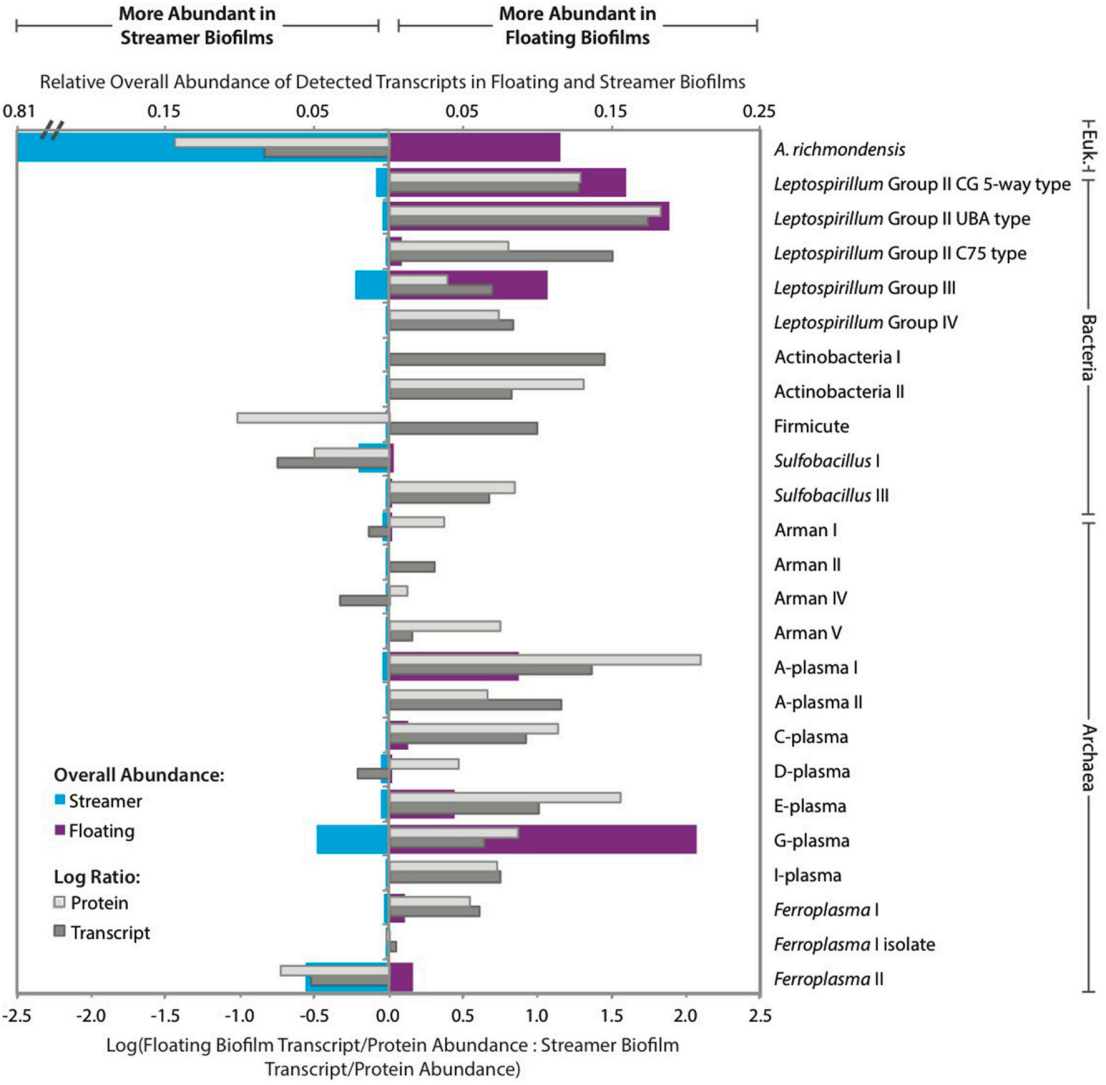
**Relative abundance of detected transcripts and proteins per organism in floating and streamer biofilms**. The log ratio of transcript (dark gray) and protein (light gray) abundance between floating and streamer biofilms is based on the summed transcript and protein abundance for each organism averaged across replicates. Blue- and purple-shaded bars indicate relative levels of overall transcript abundance on a linear scale.

We evaluated the community expression profile within the biofilms using transcripts and proteins quantified from 25 different bacterial, archaeal, and eukaryal organisms (Figure 3; Supplemental Figure S2). Rank abundance curves based on transcript and protein counts indicated that transcripts and proteins were unevenly distributed among organisms in streamer biofilms (Supplemental Figure S3). *A. richmondensis* transcripts and proteins dominated the streamer biofilms, averaging 85% of the community, relative to 7% of the floating community (based on transcript and protein counts). Overall, only three organisms consistently had more transcripts and proteins in the streamer biofilms than in the floating biofilms: *A. richmondensis, Sulfobacillus* I, and *Ferroplasma* II (Figure 3). Both *Sulfobacillus* and *Ferroplasma* have closely related genotypes that had more abundant transcripts and proteins in floating biofilms.

Transcript and protein abundance was more evenly distributed in floating biofilms (based on rank abundance curves; Supplemental Figure S3). In the floating biofilms, the *Leptospirillum* bacterial genotypes together accounted for 41–52% of the transcriptome and 72–80% of the proteome. Transcripts and proteins from the *Leptospirillum* bacterial genotypes were all more abundant in floating biofilms than in streamer biofilms, however, the ratio of group III to group II was lower in floating biofilms (i.e., group III were less abundant than group II in floating biofilms but more abundant in streamer biofilms).

Overall, detected transcript and protein counts are influenced by a complex combination of factors (including variations in genome copy number, gene copy number, genome size, gene size, cell count, cell volume, activity levels per cell, and inherent methodological biases) and should not be interpreted as a measure of cellular abundance.

### Expression of *A. richmondensis* genes in floating and streamer biofilms

We evaluated the predicted metabolism and *in situ* expression of *A. richmondensis* in floating and streamer biofilms. Transcript and protein abundance was evaluated at the organism-level to adjust for differences in organism abundance across samples (i.e., by making the total sum of all *A. richmondensis* transcript and protein counts identical across all samples; organism-level transcripts originated from the poly-A selected RNA sequencing libraries). A total of 9867 *A. richmondensis* transcripts quantified in all of the five biofilm replicates were considered for gene set analyses of the biofilms. More than 60 different gene sets were evaluated covering a broad range of functionality, as described below. *A. richmondensis* proteins were not considered for gene set analyses of the biofilms because only 357 *A. richmondensis* proteins were quantified in floating biofilms, due to the organism's low abundance in the floating biofilm community. Detected *A. richmondensis* proteins were, however, used to confirm expression of certain genes and pathways (see below).

### General metabolism

Transcripts involved in amino acid metabolism (including 9 KEGG pathways, such as histidine metabolism) were significantly up-regulated in streamer biofilms. Conversely, transcripts of amino acid transporters were significantly up-regulated in floating biofilms. Transcript abundance in broad metabolic pathways varied between biofilm conditions: replication, recombination, and repair; RNA processing and modification; and transcription were significantly more abundant in floating biofilms, whereas translation; ribosomal proteins; energy metabolism; and lipid metabolism were all significantly more abundant in streamer biofilms. Cytochrome *c* oxidase genes (aerobic respiration) were detected at the transcript and protein level in both types of biofilms. Nearly 100 vesicle-related genes were predicted in the *A. richmondensis* genome, and these transcripts were significantly more abundant in streamer biofilms. More than half of the vesicle-related genes were detected as proteins within the streamer biofilms, and three were detected in floating biofilms.

### Heavy metal transport and detoxification

*A. richmondensis* encoded and expressed many genes related to heavy metal transport and detoxification. The *A. richmondensis* genome had a wide range of metal transporters specific to iron, copper, zinc, magnesium, calcium, and nickel/cobalt. Several genes potentially involved in chelation were identified in the *A. richmondensis* genome, including a ferrochelatase, a siderophore dependent iron transporter, sideroflexin, and several transcripts for ferric-chelate reductase. Cyanide hydratase/nitrilase and cyanate lyase genes may act to detoxify exogenous cyanide or cyanide byproducts of other cellular metabolic reactions.

*A. richmondensis* encoded many genes for arsenic detoxification, including a putative arsenate reductase (arsH, an NADPH-dependent FMN reductase), membrane-associated arsenite permeases (ArsB or ACR3), and arsenite-translocating ATPases (ArsA). Other genes for arsenate modification included an arsenite methyltransferase, several genes with methylarsonite methyltransferase activity, and several genes encoding glutathione S-transferase, a protein implicated in reduction of arsenate to arsenite (Zakharyan et al., 2005; Ventura-Lima et al., 2011).

When considering all of the metal transport and detoxification genes together (i.e., as one gene set), metal-related transcripts were significantly up-regulated in streamer biofilms. Additionally, an individual arsenate reductase was significantly up-regulated at the protein level in streamer biofilms. Overall, 24 metal transport and detoxification proteins were measured only in streamer biofilms, and an additional eight proteins were measured in both floating and streamer biofilms.

### Compatible solute metabolism

The *A. richmondensis* genome encoded genes involved in the metabolism of compatible solutes that may be particularly useful in the high-ionic-strength waters within the Richmond Mine (Druschel et al., 2004). The *A. richmondensis* genome contained genes involved in the biosynthesis and degradation of taurine, and prior metabolomic analyses have identified taurine in AMD biofilms (Mosier et al., 2013). *A. richmondensis* also had genes involved in the biosynthesis and degradation of trehalose, as well as a betaine-aldehyde dehydrogenase gene. Overall, transcripts involved in compatible solute metabolism were significantly up-regulated in streamer biofilms. More than half of the compatible solute-related genes were measured as proteins within the streamer biofilms, and two were measured in floating biofilms.

### Carbon transformations

The *A. richmondensis* genome encoded complete TCA, glycolysis, and pentose phosphate pathways. Additionally, *A. richmondensis* contained many genes involved in the metabolism of fructose, mannose, galactose, starch, and sucrose.

The *A. richmondensis* genome was predicted to encode 350 CAZymes, which are classified as families of structurally-related enzymes that degrade, modify, or create glycosidic bonds (Lombard et al., 2014). *A. richmondensis* CAZymes included 87 glycosyl transferases (GTs; formation of glycosidic bonds), 233 glycoside hydrolases (GHs; hydrolysis/rearrangement of glycosidic bonds), 18 carbohydrate esterases (CEs; hydrolysis of carbohydrate esters), and 20 carbohydrate-binding modules (CBMs; carbohydrate-binding activity). Among the predicted CAZyme genes, 347 were quantified as transcripts and 62 were quantified as proteins. There were 45 potential cellulase genes predicted in the *A. richmondensis* genome (families GH1, GH3, GH5, GH6, GH7, GH12, GH30, GH45, GH61). Transcripts from all 45 putative cellulases were detected in the biofilms, six of which were among the top 10% of the most highly expressed transcripts. Additionally, eight putative cellulase proteins were detected in the biofilms.

**Figure 4:**
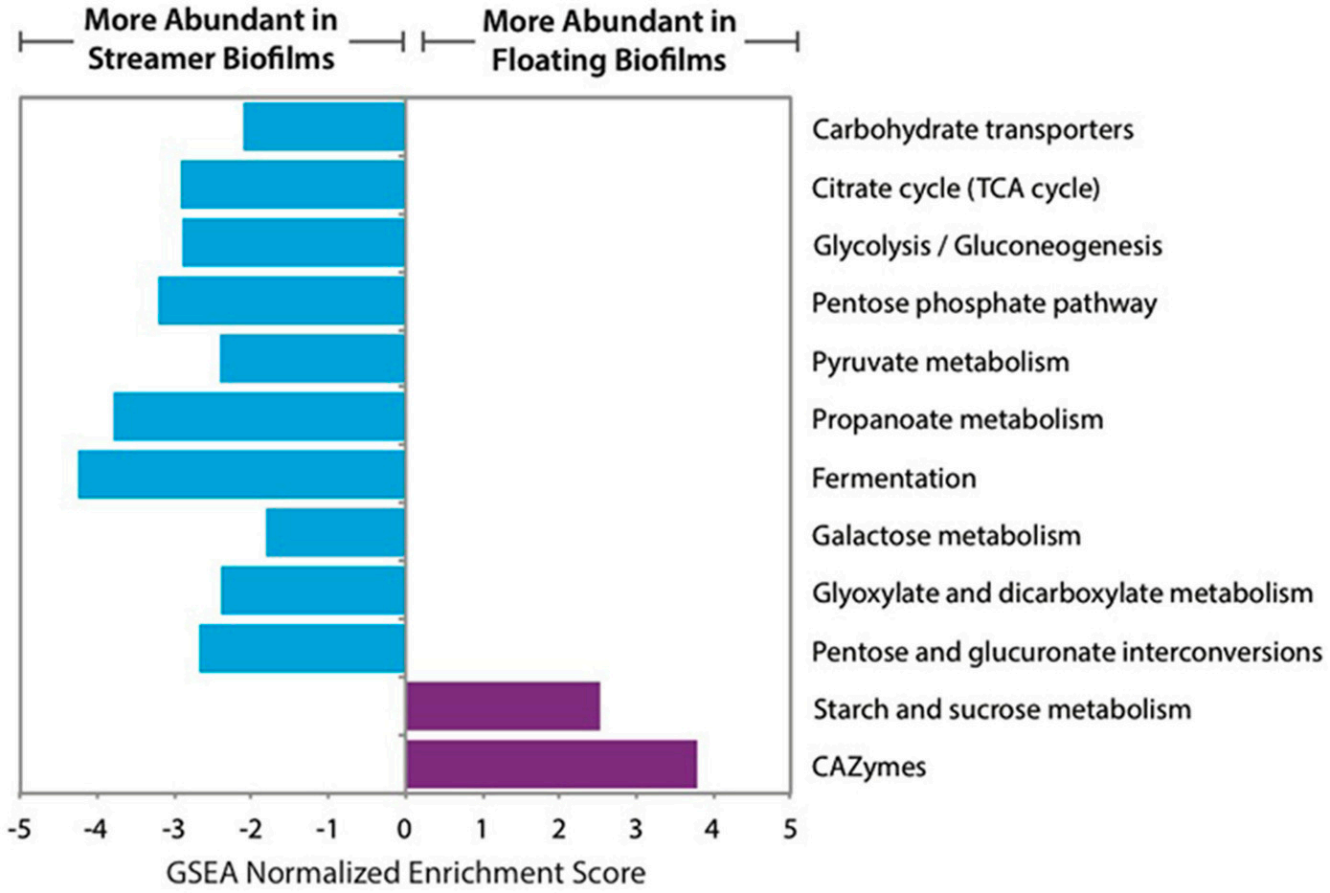
**Relative abundance of carbon-related gene sets in floating and streamer biofilms, based on GSEA normalized enrichment score of transcript abundance**. Only significant gene sets are shown (FDR *q* ≤ 0.05).

**Figure 5:**
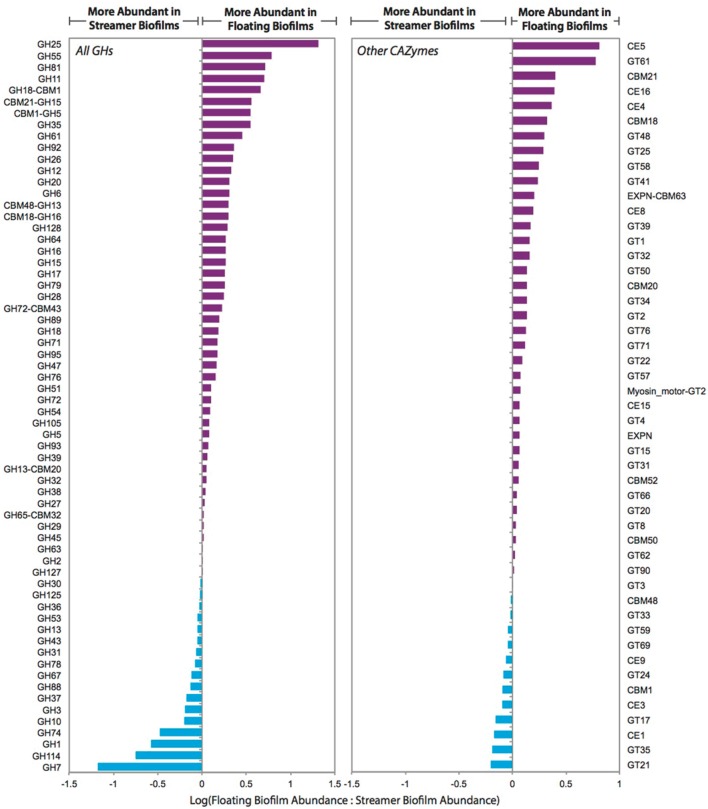
**Relative transcript abundance of carbohydrate active enzyme (CAZyme) families in floating and streamer biofilms**. Individual transcript abundances were summed for each CAZyme family across the biofilm replicates (e.g., transcripts from two genes encoding GH7s were summed for each biofilm replicate). Summed abundances were then transformed to a log ratio of the replicate average (floating biofilm average:streamer biofilm average). Only CAZyme families with transcripts measured in all five biofilm replicates are shown.

The central carbon metabolism pathways and carbohydrate transporters were significantly up-regulated in streamer biofilms (Figure 4). However, CAZymes and starch and sucrose metabolism were up-regulated in floating biofilms. CAZymes were also significantly more abundant in floating biofilms when broken down by each class (GTs, CBMs, CEs, and GHs), or when only considering cellulases, GHs acting on alpha-linked sugars, or GHs acting on beta-linked sugars. When analyzing each CAZyme family (e.g., the sum of all expressed GH88 transcripts per biofilm replicate), 75% of the families were more abundant in floating biofilms (Figure 5). At the level of individual transcripts, 16 GHs were significantly more abundant in floating biofilms, a majority of which act on beta-linked sugars (Table 2).

**Table 2:** **Glycoside Hydrolase (GH) families that were significantly different between biofilms at the level of individual transcripts (based on Rank Product fold change and ***p***-value)**.

**Greatest abundance**	**GH family**	**Substrate**	**Alpha- or Beta-linked sugars**	**Potential cellulase**
Floating biofilms	GH3	β-glucans; cellulose; xylan; peptidoglycan	Beta	Yes
	GH5	β-glucans; mannose; cellulose; xylan	Beta	Yes
	GH7	β-glucans; cellulose; xylan	Beta	Yes
	GHII	xylan	Beta	n/a
	GHI6	β-glucans and β-galactans	Beta	n/a
	GH 16	β-glucans and β-galactans	Beta	n/a
	GH25	peptidoglycan	Beta	n/a
	GH27	α-galactose; α-N-acetylgalactosamine	Alpha	n/a
	GH35	β-galactose	Beta	n/a
	GH47	α-mannose	Alpha	n/a
	GH55	β-1,3-glucan	Beta	n/a
	GH72	β-1,3-glucan	Beta	n/a
	GH78	α-rhamnose	Alpha	n/a
	GH79	β-glucuronoside	Beta	n/a
	GH81	β-1,3-glucan	Beta	n/a
	GH92	α-mannose	Alpha	n/a
Streamer biofilms	GH I	β-glucosides; β-galactose; cellulose	Beta	Yes
	GH3	β-glucans; cellulose; xylan; peptidoglycan	Beta	Yes
	GH7	β-glucans; cellulose; xylan	Beta	Yes
	GH31	α-glucans	Alpha	n/a
	GHII 4	α-galactosamine	Alpha	n/a

*A. richmondensis* encoded genes to produce acetate, ethanol, lactate, and propionate as fermentation end products. The fermentative pathway transcripts were significantly up-regulated in streamer biofilms (Figure 4). Nearly 75% of the fermentation-related genes were measured as proteins within either the floating or streamer biofilms.

### Nitrogen transformations

The *A. richmondensis* genome had genes for denitrification (the reduction of nitrate to nitrogen gasses) including a nitrate transporter, two nitrate reductases (nitrate to nitrite), a copper-containing nitrite reductase (NirK; nitrite to nitric oxide), and a cytochrome-P450nor nitric oxide reductase (nitric oxide to nitrous oxide). Each of these genes were expressed in the biofilms at the transcript level, as well as nitrite reductase proteins. Nitrous oxide reductase genes (nitrous oxide to N_2_) were not evident in the genome, similar to all known denitrifying fungi (Shoun et al., 2012). Nitrous oxide production (~6 ppm) was measured in pure cultures of *A. richmondensis* grown at pH 1 under oxic conditions where the media and headspace were aerobic but the supply of air was discontinued after the serum vials were sealed. No nitrous oxide production was detected in anaerobic cultures or in abiotic controls. Denitrification transcripts were up-regulated in fungi growing in the floating biofilms, relative to streamer biofilms.

The nitrate reductase and an assimilatory nitrite reductase (nitrite to ammonia) may be used for assimilatory or dissimilatory purposes via ammonia fermentation as seen with the fungus *Fusarium oxysporum* (Zhou et al., 2002). *A. richmondensis* also had ammonium transporters and urease genes, which facilitate the hydrolysis of urea into carbon dioxide and ammonia.

## Discussion

### Reconstruction of eukaryotic genomes from environmental samples

Reconstructing eukaryotic genomes from metagenomic sequences has historically been a significant challenge and has precluded genome-based expression studies of eukaryotes within their natural habitats. Here, we reconstructed the near-complete genome of *A. richmondensis* from AMD biofilms. We applied genome assembly approaches (e.g., stepwise assembly and ESOM-based binning) that have been shown to facilitate reconstruction of complete and essentially-complete bacterial and archaeal genomes from complex samples (e.g., Dick et al., 2009; Sharon and Banfield, 2013). Our genome assembly efforts were feasible due in part to the low diversity of the eukaryotic AMD community, the small size of the targeted genome (27 Mbp), and the sequencing of the isolate genome. We hypothesize that assembly approaches such as these, together with continued developments in sequencing technologies and binning and assembly algorithms, may make eukaryotic genome reconstruction more tractable in complex systems.

### The overall distribution of transcripts and proteins among fungal, bacterial, and archaeal community members

The overall distribution of transcripts and proteins among microbial community members differed greatly between floating and streamer biofilms. *A. richmondensis* transcripts and proteins dominated the streamer biofilms (Figure 3) and comprised a significant portion of the total biomass (Figure 1). Conversely, *A. richmondensis* transcripts and proteins were at low abundance (7%) in the floating biofilms, which were dominated by *Leptospirillum* bacterial transcripts and proteins. Consistent with many studies of floating biofilms in this mine (e.g., Mueller et al., 2010; Justice et al., 2012), *Leptospirillum* group II transcripts and proteins were more abundant than group III in floating biofilms. Conversely, *Leptospirillum* group III, which can be quite abundant in some laboratory-cultivated biofilms (Belnap et al., 2011; Mosier et al., 2013, 2015), transcripts and proteins were more abundant than Group II in streamer biofilms.

Other than *A. richmondensis*, only two organisms consistently had more abundant transcripts and proteins in the streamer biofilms than in the floating biofilms: *Sulfobacillus* I and *Ferroplasma* II (Figure 3). Interestingly, both *Sulfobacillus* and *Ferroplasma* had closely related genotypes that had more abundant transcripts and proteins in floating biofilms (*Sulfobacillus* III and *Ferroplasma* I), which may be suggestive of niche differentiation within these groups. *Sulfobacillus* species are facultative anaerobic autotrophs, and genomic differences between types I and III include genes involved in sulfur metabolism, carbon monoxide oxidation, and nitrate reduction (Justice et al., 2014). Prior genomic analyses indicated that both *Ferroplasma* type I and II are facultative aerobic heterotrophs and that differences between the strains included genes involved in carbon monoxide oxidation, mercury resistance, and S-layer structure (Yelton et al., 2013). It is possible that these genomic differences play a role in determining species distribution in floating and streamer biofilms. *Sulfobacillus* and *Ferroplasma* distribution may also be linked to fungal abundance.

### Response to environmental stresses

The chemical and physical conditions of the AMD are expected to be similar in the floating and streamer biofilm habitats, with low pH, high metal concentrations and high ionic strength. However, *A. richmondensis* genes involved in responding to these harsh conditions (i.e., metal transport and detoxification and compatible solute metabolism) were more highly expressed in streamer biofilms than in floating biofilms. Thus, *A. richmondensis* may experience greater stress in streamer biofilms. Mature biofilms floating at the air-solution interface, possibly buoyed by accumulated gases, may exchange solution slowly with underlying AMD. This may protect organisms encased in the biofilm matrix from environmental challenges. In contrast, submerged streamer biofilms are likely permeated by the flowing acidic, metal-rich solution, making this habitat considerably more challenging to the organisms within it.

### Denitrification

Denitrification by fungi has been shown in pure cultures growing at pH values ranging from ~5–7 (Shoun et al., 2012; Jasrotia et al., 2014; Rohe et al., 2014a,b). Denitrification attributed to fungi and/or archaea (i.e., denitrification as measured in the presence of bacterial inhibitors) was measured in groundwater sediments with pH as low as 3.67 (Jasrotia et al., 2014). Here, we show that *A. richmondensis* may denitrify at pH 1 (possibly the lowest recorded pH for denitrification by any bacteria, archaea, or eukaryote): *A. richmondensis* has denitrification genes for converting nitrate to nitrous oxide gas and expresses these genes as transcripts and proteins in natural biofilms. Nitrous oxide was produced in *A. richmondensis* cultures, but not in abiotic controls with nitrate. These results do not rule out the possibility of chemodenitrification (the abiotic conversion of nitrite or nitric oxide to nitrous oxide), but do show that *A. richmondensis* may play a role in nitrogen gas production (either by coupling biotic nitrate reduction to abiotic chemodenitrification; or by biotic denitrification).

Denitrification is predominantly performed by facultative anaerobes in anoxic environments such as saturated soils, estuary sediments, and wetlands (e.g., Prieme et al., 2002; Henry et al., 2004; Seitzinger et al., 2006; Mosier and Francis, 2010). For *A. richmondensis*, nitrous oxide production occurred in aerobic cultures, but not in anaerobic cultures. Aerobic cultures were grown in sealed vials where the media and headspace were aerobic but no additional air was supplied during the growth experiment. Aerobic respiration likely decreased oxygen concentrations in the culture vials over time. Other fungal cultures have also shown that denitrifying activity requires a minimal amount of oxygen (Zhou et al., 2001; Jasrotia et al., 2014). This finding is consistent with the observation that *A. richmondensis* denitrification transcripts were up-regulated in floating biofilms, which have been shown to have low oxygen concentrations in the interior biofilm regions due to diffusion limitation (Ma and Banfield, 2011). Taken together, we infer that denitrification by *A. richmondensis* may be most important in microaerophilic conditions.

Interestingly, a few organisms are able to simultaneously use both oxygen and nitrate, though with lower productivity than when using oxygen alone (Robertson and Kuenen, 1984; Tanimoto et al., 1992). *A. richmondensis* denitrification and cytochrome *c* oxidase (aerobic respiration) transcripts and proteins were simultaneously quantified in floating and streamer biofilms. This may suggest that *A. richmondensis* constitutively expresses transcripts for both aerobic respiration and for denitrification in order to adapt to fluctuating conditions or that micro-niches are present within each habitat.

### Carbon transformations

*A. richmondensis* CAZymes were up-regulated in floating biofilms, including those that hydrolyze polymers of the sugars glucose, mannose, xylose, rhamnose, and galactose. Many of these sugars have been previously shown to be constituents of the extracellular matrix in the AMD floating biofilms (Jiao et al., 2010). These sugars may be synthesized by the bacterial and archaeal biofilm community members. For instance, *Leptospirillum* bacteria (the dominant members of floating biofilms) have genes for cellulose biosynthesis (Aliaga Goltsman et al., 2009, 2013) and *Ferroplasma* type II have genes for generating galactose. Some GHs involved in the degradation of peptidoglycan (a major membrane component of bacteria) were also up-regulated in floating biofilms. Overall, this may suggest that *A. richmondensis* utilizes complex carbon sources accessed from its close interactions with other community members in the floating biofilms.

In streamer biofilms, central carbon metabolism and carbohydrate transporters were significantly up-regulated relative to floating biofilms. This might suggest that *A. richmondensis* utilizes simple carbon sources dissolved within the stream, since it does not have access to the reservoir of complex carbon within the floating biofilms.

## Conclusions

We reconstructed the near-complete genome of a microbial eukaryote, *A. richmondensis*, from a metagenomics dataset and confirmed the genome by comparison with that of an isolate. By examining the differential gene expression of this fungus in two distinct types of biofilm communities, we identified differences in its metabolic functioning, particularly with regard to carbon and nitrogen substrate utilization. The functional differences may be due in part to the differences in the composition and metabolism of the associated microbial community and they may reflect distinct physical and chemical conditions in the two niches. The results of this study further establish the value of linked metagenomic, transcriptomic, and proteomic approaches for prediction and functional analysis of the roles of microbes in natural ecosystems.

## Author contributions

Conceived and designed the experiments: AM, CM, CP, SS, JB. Performed the experiments: AM, CM, KF, RO, ZL, CC, JK. Analyzed the data: AM, CM, KF, RO, ZL, KL, ALa, ALi. Contributed to the manuscript: AM, CM, KF, RO, ZL, EL, CP, IG, SS, JB. Funded the research: AM, CM, CP, RH, SS, JB.

### Conflict of interest statement

The authors declare that the research was conducted in the absence of any commercial or financial relationships that could be construed as a potential conflict of interest.

## References

[B1] Albertsen M. Hugenholtz P. Skarshewski A. Nielsen K. L. Tyson G. W. Nielsen P. H. (2013). Genome sequences of rare, uncultured bacteria obtained by differential coverage binning of multiple metagenomes. Nat. Biotechnol. 31, 533–538. 10.1038/nbt.2579 23707974

[B2] Aliaga Goltsman D. S. Dasari M. Thomas B. C. Shah M. B. VerBerkmoes N. C. Hettich R. L.. (2013). New group in the leptospirillum clade: cultivation-independent community genomics, proteomics, and transcriptomics of the new species “Leptospirillum Group IV UBA BS.” Appl. Environ. Microbiol. 79, 5384–5393. 10.1128/AEM.00202-13 23645189 PMC3753937

[B3] Aliaga Goltsman D. S. Denef V. J. Singer S. W. VerBerkmoes N. C. Lefsrud M. Mueller R. S.. (2009). Community genomic and proteomic analyses of chemoautotrophic iron-oxidizing “Leptospirillum rubarum” (Group II) and “Leptospirillum ferrodiazotrophum” (Group III) bacteria in acid mine drainage biofilms. Appl. Environ. Microbiol. 75, 4599–4615. 10.1128/AEM.02943-08 19429552 PMC2704813

[B4] Altschul S. F. Gish W. Miller W. Myers E. W. Lipman D. J. (1990). Basic local alignment search tool. J. Mol. Biol. 215, 403–410. 10.1016/S0022-2836(05)80360-2 2231712

[B5] Altschul S. F. S. Madden T. L. T. Schäffer A. A. A. Zhang J. J. Zhang Z. Z. Miller W. W.. (1997). Gapped BLAST and PSI-BLAST: a new generation of protein database search programs. Nucleic Acids Res. 25, 3389–3402. 10.1093/nar/25.17.3389 9254694 PMC146917

[B6] Amann R. I. Ludwig W. Schleifer K. H. (1995). Phylogenetic identification and *in situ* detection of individual microbial cells without cultivation. Microbiol. Rev. 59, 143–169. 7535888 10.1128/mr.59.1.143-169.1995 PMC239358

[B7] Ashburner M. Ball C. A. Blake J. A. Botstein D. Butler H. Cherry J. M.. (2000). Gene ontology: tool for the unification of biology. The gene ontology consortium. Nat. Genet. 25, 25–29. 10.1038/75556 10802651 PMC3037419

[B8] Baker B. J. Banfield J. F. (2003). Microbial communities in acid mine drainage. FEMS Microbiol. Ecol. 44, 139–152. 10.1016/S0168-6496(03)00028-X 19719632

[B9] Baker B. J. Lutz M. A. Dawson S. C. Bond P. L. Banfield J. F. (2004). Metabolically active eukaryotic communities in extremely acidic mine drainage. Appl. Environ. Microbiol. 70, 6264–6271. 10.1128/AEM.70.10.6264-6271.2004 15466574 PMC522060

[B10] Baker B. J. Tyson G. W. Goosherst L. Banfield J. F. (2009). Insights into the diversity of eukaryotes in acid mine drainage biofilm communities. Appl. Environ. Microbiol. 75, 2192–2199. 10.1128/AEM.02500-08 19201960 PMC2663193

[B11] Belnap C. P. Pan C. Denef V. J. Samatova N. F. Hettich R. L. Banfield J. F. (2011). Quantitative proteomic analyses of the response of acidophilic microbial communities to different pH conditions. ISME J. 5, 1152–1161. 10.1038/ismej.2010.200 21228889 PMC3146278

[B12] Bond P. Banfield J. (2001). Design and performance of rRNA targeted oligonucleotide probes for *in situ* detection and phylogenetic identification of microorganisms inhabiting acid mine drainage environments. Microb. Ecol. 41, 149–161. 10.1007/s002480000063 12032620

[B13] Breitling R. Armengaud P. Amtmann A. Herzyk P. (2004). Rank products: a simple, yet powerful, new method to detect differentially regulated genes in replicated microarray experiments. FEBS Lett. 573, 83–92. 10.1016/j.febslet.2004.07.055 15327980

[B14] Cantarel B. L. Coutinho P. M. Rancurel C. Bernard T. Lombard V. Henrissat B. (2009). The Carbohydrate-Active EnZymes database (CAZy): an expert resource for Glycogenomics. Nucleic Acids Res. 37, D233–D238. 10.1093/nar/gkn663 18838391 PMC2686590

[B15] Cantu D. Govindarajulu M. Kozik A. Wang M. Chen X. Kojima K. K.. (2011). Next generation sequencing provides rapid access to the genome of *Puccinia striiformis* f. sp. tritici, the causal agent of wheat stripe rust. PLoS ONE 6:e24230. 10.1371/journal.pone.0024230 21909385 PMC3164196

[B16] Castelle C. J. Hug L. A. Wrighton K. C. Thomas B. C. Williams K. H. Wu D.. (2013). Extraordinary phylogenetic diversity and metabolic versatility in aquifer sediment. Nat. Commun. 4, 2120. 10.1038/ncomms3120 23979677 PMC3903129

[B17] Chourey K. Jansson J. VerBerkmoes N. Shah M. Chavarria K. L. Tom L. M.. (2010). Direct cellular lysis/protein extraction protocol for soil metaproteomics. J. Proteome Res. 9, 6615–6622. 10.1021/pr100787q 20954746

[B18] Cissé O. H. Pagni M. Hauser P. M. (2012). *De novo* assembly of the *Pneumocystis jirovecii* genome from a single bronchoalveolar lavage fluid specimen from a patient. MBio 4, e00428–12. 10.1128/mBio.00428-12 23269827 PMC3531804

[B19] Cuvelier M. L. Allen A. E. Monier A. McCrow J. P. Messié M. Tringe S. G.. (2010). Targeted metagenomics and ecology of globally important uncultured eukaryotic phytoplankton. Proc. Natl. Acad. Sci. U.S.A. 107, 14679–14684. 10.1073/pnas.1001665107 20668244 PMC2930470

[B20] Denef V. J. Mueller R. S. Banfield J. F. (2010). AMD biofilms: using model communities to study microbial evolution and ecological complexity in nature. ISME J. 4, 599–610. 10.1038/ismej.2009.158 20164865

[B21] Di Rienzi S. C. Sharon I. Wrighton K. C. Koren O. Hug L. A. Thomas B. C.. (2013). The human gut and groundwater harbor non-photosynthetic bacteria belonging to a new candidate phylum sibling to Cyanobacteria. Elife 2:e01102. 10.7554/elife.01102 24137540 PMC3787301

[B22] Dick G. J. Andersson A. F. Baker B. J. Simmons S. L. Thomas B. C. Yelton A. P.. (2009). Community-wide analysis of microbial genome sequence signatures. Genome Biol. 10:R85. 10.1186/gb-2009-10-8-r85 19698104 PMC2745766

[B23] Dobbin E. Graham C. Freeburn R. W. Unwin R. D. Griffiths J. R. Pierce A.. (2010). Proteomic analysis reveals a novel mechanism induced by the leukemic oncogene Tel/PDGFRβ in stem cells: activation of the interferon response pathways. Stem Cell Res. 5, 226–243. 10.1016/j.scr.2010.08.001 20875954

[B24] Druschel G. Baker B. Gihring T. Banfield J. (2004). Acid mine drainage biogeochemistry at Iron Mountain, California. Geochem. Trans. 5, 13–32. 10.1186/1467-4866-5-13 PMC1475782 35412773

[B25] Eddy S. R. (2009). A new generation of homology search tools based on probabilistic inference. Genome Inform. 23, 205–211. 10.1142/9781848165632_0019 20180275

[B26] Enright A. J. Van Dongen S. Ouzounis C. A. (2002). An efficient algorithm for large-scale detection of protein families. Nucleic Acids Res. 30, 1575–1584. 10.1093/nar/30.7.1575 11917018 PMC101833

[B27] Grigoriev I. V. Nikitin R. Haridas S. Kuo A. Ohm R. Otillar R.. (2014). MycoCosm portal: gearing up for 1000 fungal genomes. Nucleic Acids Res. 42, D699–D704. 10.1093/nar/gkt1183 24297253 PMC3965089

[B28] Grigoriev I. V. Nordberg H. Shabalov I. Aerts A. Cantor M. Goodstein D.. (2012). The genome portal of the Department of Energy Joint Genome Institute. Nucleic Acids Res. 40, D26–D32. 10.1093/nar/gkr947 22110030 PMC3245080

[B29] Han D. Moon S. Kim H. Choi S.-E. Lee S.-J. Park K. S.. (2011). Detection of differential proteomes associated with the development of type 2 diabetes in the Zucker rat model using the iTRAQ technique. J. Proteome Res. 10, 564–577. 10.1021/pr100759a 21117707

[B30] Henry S. Baudoin E. López-Gutiérrez J. C. Martin-Laurent F. Brauman A. Philippot L. (2004). Quantification of denitrifying bacteria in soils by nirK gene targeted real-time PCR. J. Microbiol. Methods 59, 327–335. 10.1016/j.mimet.2004.07.002 15488276

[B31] Hujslová M. Kubátová A. Chudíèková M. Kolaøík M. (2009). Diversity of fungal communities in saline and acidic soils in the Soos National Natural Reserve, Czech Republic. Mycol Progress 9, 1–15. 10.1007/s11557-009-0611-7

[B32] Hujslová M. Kubátová A. Kostovèík M. Kolaøík M. (2013). *Acidiella bohemica* gen. et sp. nov. and Acidomyces spp. (Teratosphaeriaceae), the indigenous inhabitants of extremely acidic soils in Europe. Fungal Divers. 58, 33–45. 10.1007/s13225-012-0176-7

[B33] Hunter S. Apweiler R. Attwood T. K. Bairoch A. Bateman A. Binns D.. (2009). InterPro: the integrative protein signature database. Nucleic Acids Res. 37, D211–D215. 10.1093/nar/gkn785 18940856 PMC2686546

[B34] Hyatt D. D. Pan C. C. (2012). Exhaustive database searching for amino acid mutations in proteomes. Bioinformatics 28, 1895–1901. 10.1093/bioinformatics/bts274 22581177

[B35] Iverson V. Morris R. M. Frazar C. D. Berthiaume C. T. Morales R. L. Armbrust E. V. (2012). Untangling genomes from metagenomes: revealing an uncultured class of marine euryarchaeota. Science 335, 587–590. 10.1126/science.1212665 22301318

[B36] Jain S. Graham C. Graham R. L. J. McMullan G. Ternan N. G. (2011). Quantitative proteomic analysis of the heat stress response in Clostridium difficile strain 630. J. Proteome Res. 10, 3880–3890. 10.1021/pr200327t 21786815

[B37] Jasrotia P. Green S. J. Canion A. Overholt W. A. Prakash O. Wafula D.. (2014). Watershed-scale fungal community characterization along a pH gradient in a subsurface environment cocontaminated with uranium and nitrate. Appl. Environ. Microbiol. 80, 1810–1820. 10.1128/AEM.03423-13 24389927 PMC3957637

[B38] Jiao Y. Cody G. D. Harding A. K. Wilmes P. Schrenk M. Wheeler K. E.. (2010). Characterization of extracellular polymeric substances from acidophilic microbial biofilms. Appl. Environ. Microbiol. 76, 2916–2922. 10.1128/AEM.02289-09 20228116 PMC2863431

[B39] Justice N. B. Norman A. Brown C. T. Singh A. Thomas B. C. Banfield J. F. (2014). Comparison of environmental and isolate Sulfobacillus genomes reveals diverse carbon, sulfur, nitrogen, and hydrogen metabolisms. BMC Genomics 15:1107. 10.1186/1471-2164-15-1107 25511286 PMC4378227

[B40] Justice N. B. Pan C. Mueller R. Spaulding S. E. Shah V. Sun C. L.. (2012). Heterotrophic archaea contribute to carbon cycling in low-pH, Suboxic biofilm communities. Appl. Environ. Microbiol. 78, 8321–8330. 10.1128/AEM.01938-12 23001646 PMC3497393

[B41] Kanehisa M. Araki M. Goto S. Hattori M. Hirakawa M. Itoh M.. (2008). KEGG for linking genomes to life and the environment. Nucleic Acids Res. 36, D480–D484. 10.1093/nar/gkm882 18077471 PMC2238879

[B42] Kantor R. S. Wrighton K. C. Handley K. M. Sharon I. Hug L. A. Castelle C. J.. (2013). Small genomes and sparse metabolisms of sediment-associated bacteria from four candidate phyla. MBio 4, e00708–e00713. 10.1128/mbio.00708-13 24149512 PMC3812714

[B43] Katoh K. Standley D. M. (2013). MAFFT multiple sequence alignment software version 7: improvements in performance and usability. Mol. Biol. Evol. 30, 772–780. 10.1093/molbev/mst010 23329690 PMC3603318

[B44] Koonin E. V. Fedorova N. D. Jackson J. D. Jacobs A. R. Krylov D. M. Makarova K. S.. (2004). A comprehensive evolutionary classification of proteins encoded in complete eukaryotic genomes. Genome Bio 5:R7. 10.1186/gb-2004-5-2-r7 14759257 PMC395751

[B45] Langmead B. Trapnell C. Pop M. Salzberg S. L. (2009). Ultrafast and memory-efficient alignment of short DNA sequences to the human genome. Genome Biol. 10:R25. 10.1186/gb-2009-10-3-r25 19261174 PMC2690996

[B46] Li H. Durbin R. (2009). Fast and accurate short read alignment with Burrows-Wheeler transform. Bioinformatics 25, 1754–1760. 10.1093/bioinformatics/btp324 19451168 PMC2705234

[B47] Liao H. L. Chen Y. Bruns T. D. Peay K. G. Taylor J. W. Branco S.. (2014). Metatranscriptomic analysis of ectomycorrhizal roots reveals genes associated with Piloderma-Pinus symbiosis: improved methodologies for assessing gene expression *in situ*. Environ. Microbiol. 16, 3730–3742. 10.1111/1462-2920.12619 25186788

[B48] Lombard V. Ramulu H. G. Drula E. Coutinho P. M. Henrissat B. (2014). The carbohydrate-active enzymes database (CAZy) in 2013. Nucleic Acids Res. 42, D490–D495. 10.1093/nar/gkt1178 24270786 PMC3965031

[B49] Luo H. Wang Y. Wang H. Yang J. Yang Y. Huang H.. (2008). A novel highly acidic β-mannanase from the acidophilic fungus Bispora sp. MEY-1: gene cloning and overexpression in *Pichia pastoris*. Appl. Microbiol. Biotechnol. 82, 453–461. 10.1007/s00253-008-1766-x 18998121

[B50] Ma S. Banfield J. F. (2011). Micron-scale Fe2+/Fe3+, intermediate sulfur species and O2 gradients across the biofilm-solution-sediment interface control biofilm organization. Geochim. Cosmo. Acta 75, 3568–3580. 10.1016/j.gca.2011.03.035

[B51] Monier A. Welsh R. M. Gentemann C. Weinstock G. Sodergren E. Armbrust E. V.. (2012). Phosphate transporters in marine phytoplankton and their viruses: cross-domain commonalities in viral-host gene exchanges. Environ. Microbiol. 14, 162–176. 10.1111/j.1462-2920.2011.02576.x 21914098 PMC3429862

[B52] Mosier A. C. Francis C. A. (2010). Denitrifier abundance and activity across the San Francisco Bay estuary. Environ. Microbiol. Rep. 2, 667–676. 10.1111/j.1758-2229.2010.00156.x 23766254

[B53] Mosier A. C. Justice N. B. Bowen B. P. Baran R. Thomas B. C. Northen T. R.. (2013). Metabolites associated with adaptation of microorganisms to an acidophilic, metal-rich environment identified by stable-isotope-enabled metabolomics. MBio 4:e00484–12. 10.1128/mBio.00484-12 23481603 PMC3604775

[B54] Mosier A. C. Li Z. Thomas B. C. Hettich R. L. Pan C. Banfield J. F. (2015). Elevated temperature alters proteomic responses of individual organisms within a biofilm community. ISME J. 9, 180–194. 10.1038/ismej.2014.113 25050524 PMC4274423

[B55] Mueller R. S. Denef V. J. Kalnejais L. H. Suttle K. B. Thomas B. C. Wilmes P.. (2010). Ecological distribution and population physiology defined by proteomics in a natural microbial community. Mol. Syst. Biol. 6, 374. 10.1038/msb.2010.30 20531404 PMC2913395

[B56] Muthukrishnan G. Quinn G. A. Lamers R. P. Diaz C. Cole A. L. Chen S.. (2011). Exoproteome of *Staphylococcus aureus* reveals putative determinants of nasal carriage. J. Proteome Res. 10, 2064–2078. 10.1021/pr200029r 21338050 PMC3070068

[B57] Ohm R. A. Feau N. Henrissat B. Schoch C. L. Horwitz B. A. Barry K. W.. (2012). Diverse lifestyles and strategies of plant pathogenesis encoded in the genomes of eighteen dothideomycetes fungi. PLoS Pathog. 8:e1003037. 10.1371/journal.ppat.1003037 23236275 PMC3516569

[B58] Pan C. Fischer C. R. Hyatt D. Bowen B. P. Hettich R. L. Banfield J. F. (2011). Quantitative tracking of isotope flows in proteomes of microbial communities. Mol. Cell. Proteomics 10:M110.006049. 10.1074/mcp.M110.006049 21285414 PMC3069347

[B59] Pan C. Kora G. McDonald W. H. Tabb D. L. VerBerkmoes N. C. Hurst G. B.. (2006). ProRata: a quantitative proteomics program for accurate protein abundance ratio estimation with confidence interval evaluation. Anal. Chem. 78, 7121–7131. 10.1021/ac060654b 17037911

[B60] Parra G. Bradnam K. Ning Z. Keane T. Korf I. (2009). Assessing the gene space in draft genomes. Nucleic Acids Res. 37, 289–297. 10.1093/nar/gkn916 19042974 PMC2615622

[B61] Price M. N. Dehal P. S. Arkin A. P. (2010). FastTree 2 - approximately maximum-likelihood trees for large alignments. PLoS ONE 5:e9490. 10.1371/journal.pone.0009490 20224823 PMC2835736

[B62] Prieme A. Braker G. Tiedje J. (2002). Diversity of nitrite reductase (nirK and nirS) gene fragments in forested upland and wetland soils. Appl. Environ. Microbiol. 68, 1893–1900. 10.1128/AEM.68.4.1893-1900.2002 11916709 PMC123828

[B63] Punta M. Coggill P. C. Eberhardt R. Y. Mistry J. Tate J. Boursnell C.. (2011). The Pfam protein families database. Nucleic Acids Res. 40, D290–D301. 10.1093/nar/gkr1065 22127870 PMC3245129

[B64] Quandt C. A. Kohler A. Hesse C. N. Sharpton T. J. Martin F. Spatafora J. W. (2015). Metagenome sequence of *Elaphomyces granulatus* from sporocarp tissue reveals Ascomycota ectomycorrhizal fingerprints of genome expansion and a Proteobacteria-rich microbiome. Environ. Microbiol. 17, 2952–2968. 10.1111/1462-2920.12840 25753751

[B65] Robertson L. A. Kuenen J. G. (1984). Aerobic denitrification: a controversy revived. Arch. Microbiol. 139, 351–354. 10.1007/BF00408378

[B66] Rohe L. Anderson T.-H. Braker G. Flessa H. Giesemann A. Lewicka-Szczebak D.. (2014a). Dual isotope and isotopomer signatures of nitrous oxide from fungal denitrification–a pure culture study. Rapid Commun. Mass Spectrom. 28, 1893–1903. 10.1002/rcm.6975 25088133

[B67] Rohe L. Anderson T.-H. Braker G. Flessa H. Giesemann A. Wrage-Mönnig N.. (2014b). Fungal oxygen exchange between denitrification intermediates and water. Rapid Commun. Mass Spectrom. 28, 377–384. 10.1002/rcm.6790 24395505

[B68] Saeed A. I. Sharov V. White J. Li J. Liang W. Bhagabati N.. (2003). TM4: a free, open-source system for microarray data management and analysis. Biotechniques 34, 374–378. 12613259 10.2144/03342mt01

[B69] Schoch C. L. Seifert K. A. Huhndorf S. Robert V. Spouge J. L. Levesque C. A.. (2012). Nuclear ribosomal internal transcribed spacer (ITS) region as a universal DNA barcode marker for Fungi. Proc. Natl. Acad. Sci. U.S.A. 109, 6241–6246. 10.1073/pnas.1117018109 22454494 PMC3341068

[B70] Seitzinger S. Harrison J. Bohlke J. Bouwman A. Lowrance R. Peterson B.. (2006). Denitrification across landscapes and waterscapes: a synthesis. Ecol. Appl. 16, 2064–2090. 10.1890/1051-0761(2006)016[2064:DALAWA]2.0.CO;2 17205890

[B71] Selbmann L. de Hoog G. S. Zucconi L. Isola D. Ruisi S. van den Ende A. H.. (2008). Drought meets acid: three new genera in a dothidealean clade of extremotolerant fungi. Stud. Mycol. 61, 1–20. 10.3114/sim.2008.61.01 19287523 PMC2610311

[B72] Sharon I. Banfield J. F. (2013). Genomes from metagenomics. Science 342, 1057–1058. 10.1126/science.1247023 24288324

[B73] Sharon I. Morowitz M. J. Thomas B. C. Costello E. K. Relman D. A. Banfield J. F. (2013). Time series community genomics analysis reveals rapid shifts in bacterial species, strains, and phage during infant gut colonization. Genome Res. 23, 111–120. 10.1101/gr.142315.112 22936250 PMC3530670

[B74] Shoun H. Fushinobu S. Jiang L. Kim S.-W. Wakagi T. (2012). Fungal denitrification and nitric oxide reductase cytochrome P450nor. Philos. Trans. R. Soc. Lond. B Biol. Sci. 367, 1186–1194. 10.1098/rstb.2011.0335 22451104 PMC3306627

[B75] Sigler L. Carmichael J. W. (1974). A new acidophilic Scytalidium. Can. J. Microbiol. 20, 267–268. 10.1139/m74-043 4822789

[B76] Soares N. C. Cabral M. P. Gayoso C. Mallo S. Rodriguez-Velo P. Fernández-Moreira E.. (2010). Associating growth-phase-related changes in the proteome of *Acinetobacter baumannii* with increased resistance to oxidative stress. J. Proteome Res. 9, 1951–1964. 10.1021/pr901116r 20108952

[B77] Subramanian A. Tamayo P. Mootha V. K. Mukherjee S. Ebert B. L. Gillette M. A.. (2005). Gene set enrichment analysis: a knowledge-based approach for interpreting genome-wide expression profiles. Proc. Natl. Acad. Sci. U.S.A. 102, 15545–15550. 10.1073/pnas.0506580102 16199517 PMC1239896

[B78] Talavera G. Castresana J. (2007). Improvement of phylogenies after removing divergent and ambiguously aligned blocks from protein sequence alignments. Syst. Biol. 56, 564–577. 10.1080/10635150701472164 17654362

[B79] Tanimoto T. Nakahara K. Shoun H. (1992). Diauxic growth of *Fusarium oxysporum* during aerobic culture in the presence of nitrate/nitrite. Biosci. Biotechnol. Biochem. 56, 2058–2059. 10.1271/bbb.56.2058

[B80] Tatusov R. L. Fedorova N. D. Jackson J. D. Jacobs A. R. Kiryutin B. Koonin E. V.. (2003). The COG database: an updated version includes eukaryotes. BMC Bioinformatics 4:41. 10.1186/1471-2105-4-41 12969510 PMC222959

[B81] Tatusov R. L. Koonin E. V. Lipman D. J. (1997). A genomic perspective on protein families. Science 278, 631–637. 10.1126/science.278.5338.631 9381173

[B82] Vaulot D. Lepère C. Toulza E. De la Iglesia R. Poulain J. Gaboyer F.. (2012). Metagenomes of the picoalga Bathycoccus from the Chile coastal upwelling. PLoS ONE 7:e39648. 10.1371/journal.pone.0039648 22745802 PMC3382182

[B83] Ventura-Lima J. Bogo M. R. Monserrat J. M. (2011). Arsenic toxicity in mammals and aquatic animals: a comparative biochemical approach. Ecotoxicol. Environ. Saf. 74, 211–218. 10.1016/j.ecoenv.2010.11.002 21112631

[B84] Vizcaíno J. A. Csordas A. del-Toro N. Dianes J. A. Griss J. Lavidas I.. (2016). 2016 update of the PRIDE database and its related tools. Nucleic Acids Res. 44, D447–D456. 10.1093/nar/gkv1145 26527722 PMC4702828

[B85] Wang Y. Ahn T.-H. Li Z. Pan C. (2013). Sipros/ProRata: a versatile informatics system for quantitative community proteomics. Bioinformatics 29, 2064–2065. 10.1093/bioinformatics/btt329 23793753

[B86] Washburn M. P. Wolters D. Yates J. R. (2001). Large-scale analysis of the yeast proteome by multidimensional protein identification technology. Nat. Biotechnol. 19, 242–247. 10.1038/85686 11231557

[B87] Williamson A. J. K. Smith D. L. Blinco D. Unwin R. D. Pearson S. Wilson C.. (2008). Quantitative proteomics analysis demonstrates post-transcriptional regulation of embryonic stem cell differentiation to hematopoiesis. Mol. Cell. Proteomics 7, 459–472. 10.1074/mcp.M700370-MCP200 18045800

[B88] Wiśniewski J. R. Zougman A. Nagaraj N. Mann M. (2009). Universal sample preparation method for proteome analysis. Nat. Methods 6, 359–362. 10.1038/nmeth.1322 19377485

[B89] Woyke T. Tighe D. Mavromatis K. Clum A. Copeland A. Schackwitz W.. (2010). One bacterial cell, one complete genome. PLoS ONE 5:e10314. 10.1371/journal.pone.0010314 20428247 PMC2859065

[B90] Wrighton K. C. Thomas B. C. Sharon I. Miller C. S. Castelle C. J. VerBerkmoes N. C.. (2012). Fermentation, hydrogen, and sulfur metabolism in multiple uncultivated bacterial phyla. Science 337, 1661–1665. 10.1126/science.1224041 23019650

[B91] Yamazaki A. Toyama K. Nakagiri A. (2010). A new acidophilic fungus *Teratosphaeria acidotherma* (Capnodiales, Ascomycota) from a hot spring. Mycoscience 51, 443–455. 10.1007/S10267-010-0059-2

[B92] Yelton A. P. Comolli L. R. Justice N. B. Castelle C. Denef V. J. Thomas B. C.. (2013). Comparative genomics in acid mine drainage biofilm communities reveals metabolic and structural differentiation of co-occurring archaea. BMC Genomics 14:485. 10.1186/1471-2164-14-485 23865623 PMC3750248

[B93] Yoon H. S. H. Price D. C. D. Stepanauskas R. R. Rajah V. D. V. Sieracki M. E. M. Wilson W. H. W.. (2011). Single-cell genomics reveals organismal interactions in uncultivated marine protists. Science 332, 714–717. 10.1126/science.1203163 21551060

[B94] Zakharyan R. A. Tsaprailis G. Chowdhury U. K. Hernandez A. Aposhian H. V. (2005). Interactions of sodium selenite, glutathione, arsenic species, and omega class human glutathione transferase. Chem. Res. Toxicol. 18, 1287–1295. 10.1021/tx0500530 16097802

[B95] Zerbino D. R. Birney E. (2008). Velvet: algorithms for *de novo* short read assembly using de Bruijn graphs. Genome Res. 18, 821–829. 10.1101/gr.074492.107 18349386 PMC2336801

[B96] Zhao Z. Stanley B. A. Zhang W. Assmann S. M. (2010). ABA-regulated G protein signaling in Arabidopsis guard cells: a proteomic perspective. J. Proteome Res. 9, 1637–1647. 10.1021/pr901011h 20166762

[B97] Zhou Z. Takaya N. Nakamura A. Yamaguchi M. Takeo K. Shoun H. (2002). Ammonia fermentation, a novel anoxic metabolism of nitrate by fungi. J. Biol. Chem. 277, 1892–1896. 10.1074/jbc.M109096200 11713252

[B98] Zhou Z. Takaya N. Sakairi M. A. Shoun H. (2001). Oxygen requirement for denitrification by the fungus *Fusarium oxysporum*. Arch. Microbiol. 175, 19–25. 10.1007/s002030000231 11271416

