# Supplementary material for: Fungi Contribute Critical but Spatially Varying Roles in Nitrogen and Carbon Cycling in Acid Mine Drainage
Source: Front Microbiol. 2016 Mar 3;7:238. doi: 10.3389/fmicb.2016.00238 (PMC4776211; doi:10.3389/fmicb.2016.00238)

Supplemental Figure S1. Maximum likelihood phylogeny of the intergenic transcribed spacer region (ITS) of *Dothideomycetes* related to *Acidomyces richmondensis*. Nodes are labeled with local support values from 1000 resamples.

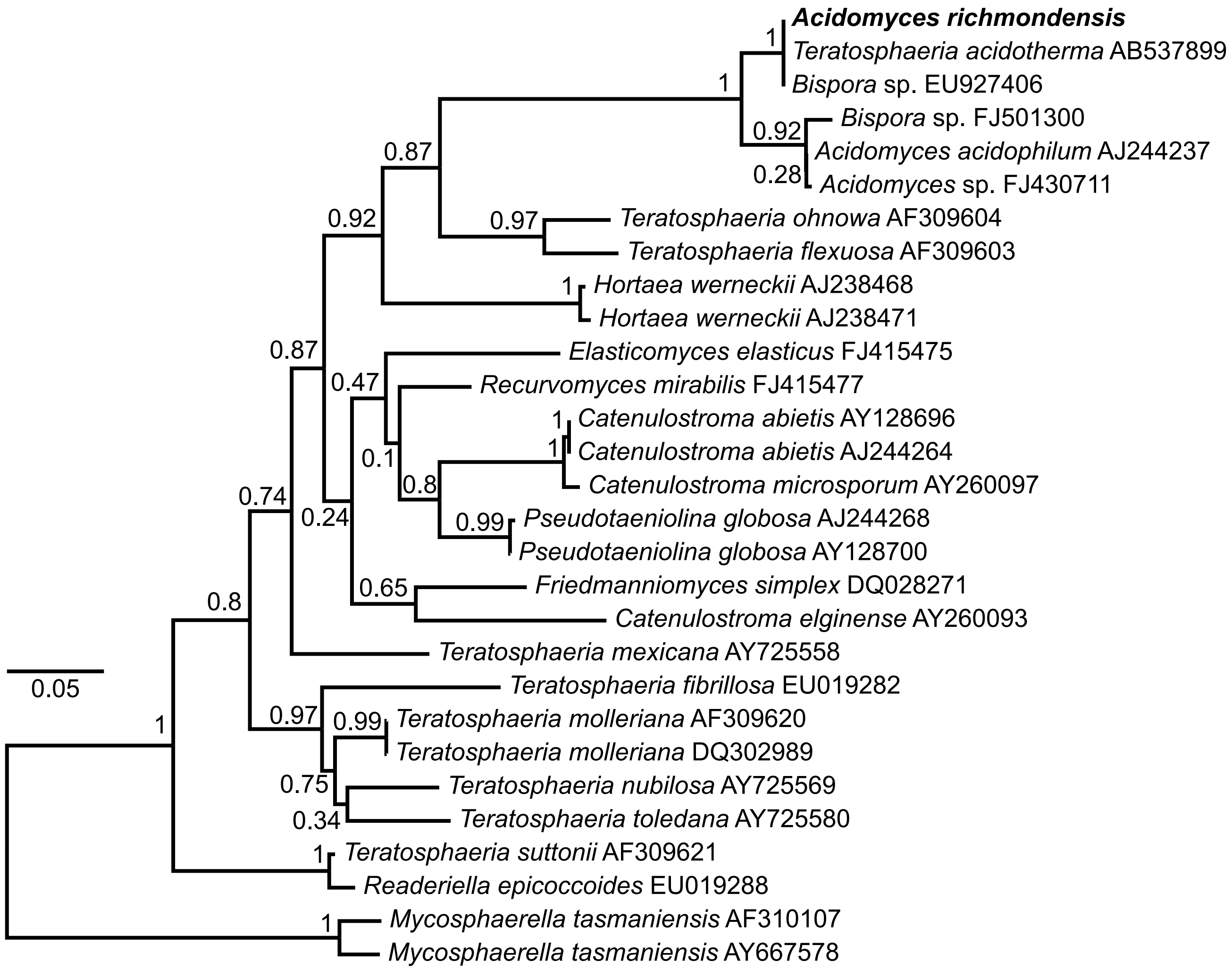

Supplement: Supplementary file 2 [file Image1.PDF]
