# Supplementary material for: Fungi Contribute Critical but Spatially Varying Roles in Nitrogen and Carbon Cycling in Acid Mine Drainage
Source: Front Microbiol. 2016 Mar 3;7:238. doi: 10.3389/fmicb.2016.00238 (PMC4776211; doi:10.3389/fmicb.2016.00238)

Supplemental Figure S2. Distribution of archaea, bacteria, and eukarya in floating and streamer biofilms based on (A) Fluorescence *In Situ* Hybridization (FISH), (B) transcript abundance, and (C) protein abundance. Transcript and protein abundance values were calculated by summing the transcript and protein counts for each organismal group (e.g., *Leptospirillum* group III, archaea, etc.) and then dividing by the total sum of all transcripts/proteins in the sample.

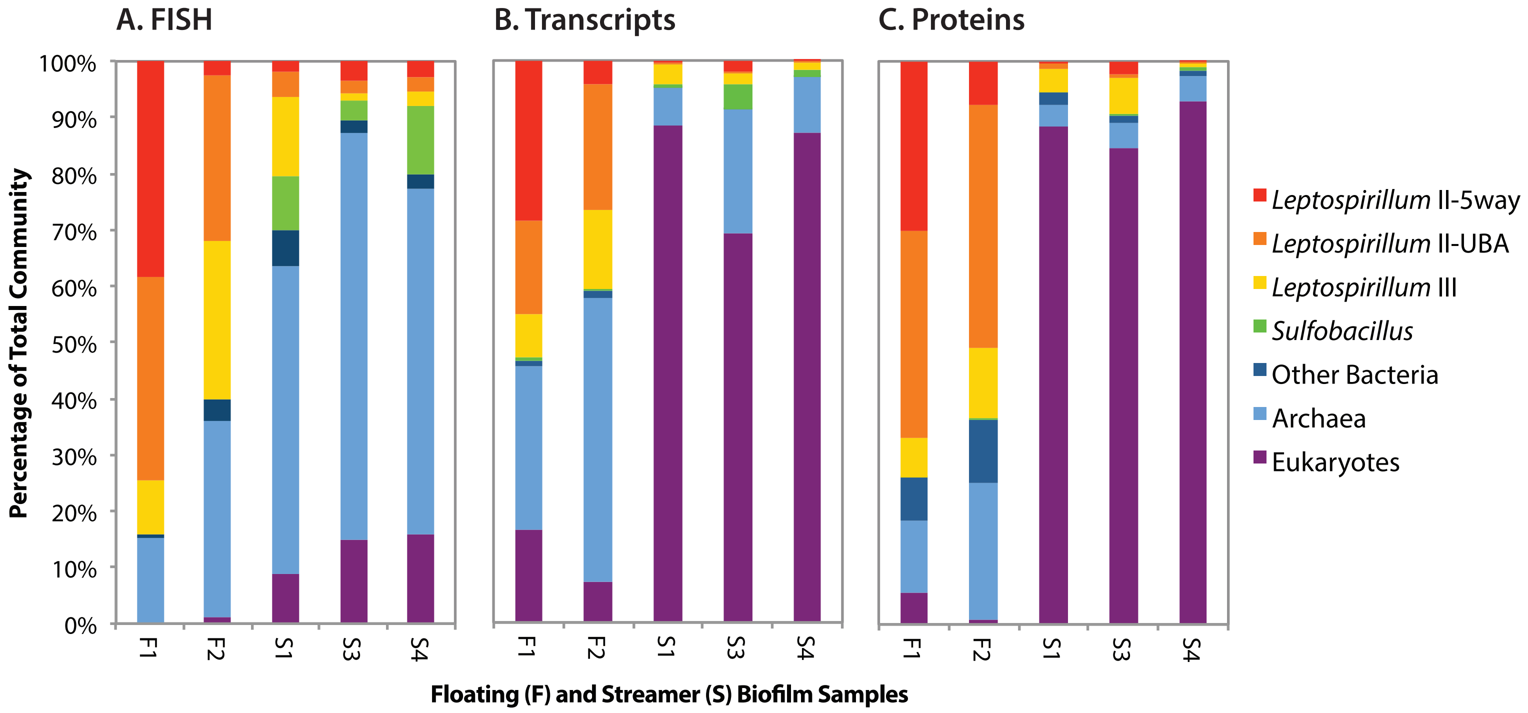

Supplement: Supplementary file 3 [file Image2.PDF]
