# Supplementary material for: Fungi Contribute Critical but Spatially Varying Roles in Nitrogen and Carbon Cycling in Acid Mine Drainage
Source: Front Microbiol. 2016 Mar 3;7:238. doi: 10.3389/fmicb.2016.00238 (PMC4776211; doi:10.3389/fmicb.2016.00238)

Supplemental Figure S3. Rank abundance of detected transcripts and proteins in floating and streamer biofilms (based on the summed transcript and protein abundance for each organism averaged across replicates).

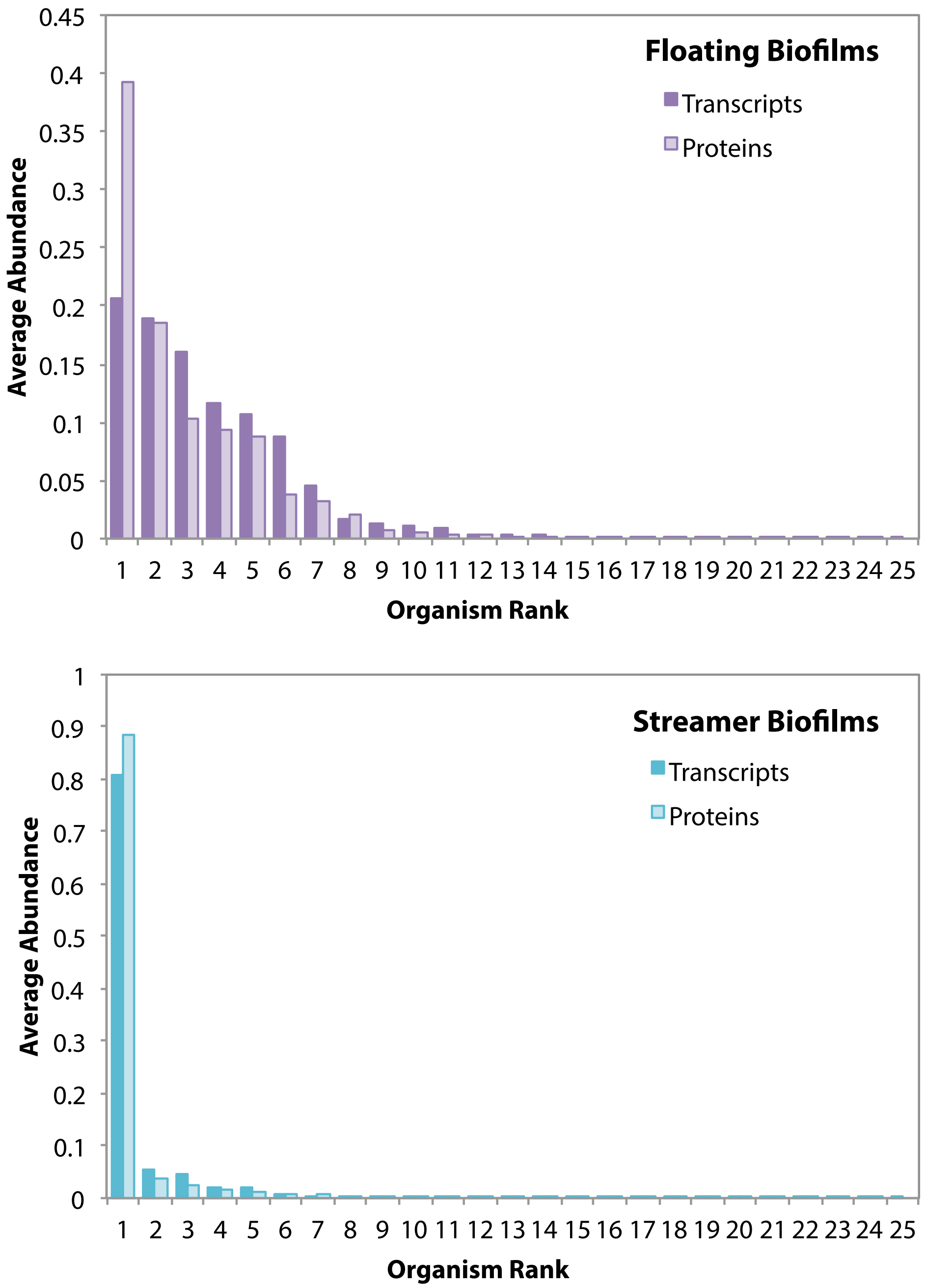

Supplement: Supplementary file 4 [file Image3.PDF]
